# A molecular ruler regulates cytoskeletal remodelling by the Rho kinases

**DOI:** 10.1038/ncomms10029

**Published:** 2015-12-01

**Authors:** Linda Truebestein, Daniel J. Elsner, Elisabeth Fuchs, Thomas A. Leonard

**Affiliations:** 1 Department of Structural and Computational Biology, Max F. Perutz Laboratories, Vienna Biocenter, 1030 Vienna, Austria; 2 Department of Medical Biochemistry, Medical University of Vienna, 1090 Vienna, Austria

## Abstract

The Rho-associated coiled-coil kinases (ROCK) are essential regulators of the actin cytoskeleton; however, the structure of a full-length ROCK is unknown and the mechanisms by which its kinase activity is controlled are not well understood. Here we determine the low-resolution structure of human ROCK2 using electron microscopy, revealing it to be a constitutive dimer, 120 nm in length, with a long coiled-coil tether linking the kinase and membrane-binding domains. We find, in contrast to previous reports, that ROCK2 activity does not appear to be directly regulated by binding to membranes, RhoA, or by phosphorylation. Instead, we show that changing the length of the tether modulates ROCK2 function in cells, suggesting that it acts as a molecular ruler. We present a model in which ROCK activity is restricted to a discrete region of the actin cytoskeleton, governed by the length of its coiled-coil. This represents a new type of spatial control, and hence a new paradigm for kinase regulation.

## Abstract (web summary)

Rho kinases regulate the actin cytoskeleton by controlling stress fibre formation. Truebestein *et al.* show that the length of its coiled-coil determines ROCK2 function, and propose that the coiled coil acts as a spacer, targeting kinase activity to a discrete distance from the membrane.

## Graphical abstract

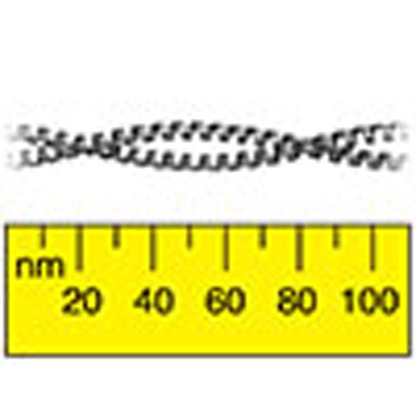

The ability of cells to change shape underpins physiological processes from embryonic development through pathogen clearance in the immune system. The plasma membrane of cells exhibits high mechanical resistance yet permits enormous changes in cell shape. This apparent paradox is resolved by a dynamic network of actin and myosin filaments beneath the plasma membrane that forms the cortical cytoskeleton. Stress fibres, bundles of actin fibres and myosin II motors traverse the cell between focal adhesions that anchor the cell in the extracellular matrix. Actomyosin contraction against these anchors generates the force required for shape change and cell motility. Phosphorylation of regulatory myosin light chain (RMLC) stimulates myosin activity, leading to contractile force generation^1^. The Rho-associated coiled-coil kinases (ROCK) are essential for the maintenance and integrity of stress fibres in the cell by directly and/or indirectly phosphorylating RMLC^2^,^3^,^4^,^5^,^6^,^7^. A null mutant of the *Drosophila* Rok gene or deletion of ROCK1 or ROCK2 in mice results in prenatal lethality^5^,^8^,^9^,^10^. The ROCK kinases comprise an N-terminal capped helix bundle domain followed by a kinase domain, central coiled-coil and C-terminal membrane-binding C1 and PH domains (Fig. 1a). The kinase domain forms a head-to-head dimer,^11^,^12^ while the PH and C1 domains are monomeric^13^. Fragments of ROCK1 and ROCK2 in the coiled-coil form parallel coiled-coils^14^,^15^,^16^,^17^, of which one has been shown to contain the Rho-binding domain^16^,^18^. RhoA activation of ROCK *in vitro*^2^,^19^,^20^,^21^ and caspase cleavage of its regulatory domains during apoptosis^22^,^23^,^24^ suggested that ROCK might be autoinhibited, analogous to the inhibition of PKC activity by its regulatory domains^25^. This concept was reinforced by the direct inhibition of kinase activity by ROCK-regulatory domains *in vitro*^26^.

We show here that ROCK2 is a constitutive parallel dimer, 120 nm in length, with N-terminal kinase domains and C-terminal regulatory domains separated by a semi-rigid coiled-coil of 107 nm. We demonstrate that, contrary to previous reports, ROCK2 activity is not influenced by its regulatory domains, RhoA, or by activation loop phosphorylation. By expressing kinase-active mutants of ROCK2 truncated in its coiled-coil, we demonstrate that, in the cellular context, constitutive ROCK2 kinase activity is regulated by the precise length of the coiled-coil that bridges the ROCK kinase domain to its substrates. We suggest a model for ROCK function in cells in which the coiled-coil acts as a molecular spacer between the membrane and the actin cytoskeleton, thereby localizing ROCK activity to a discrete subcellular environment.

## Results

### ROCK2 is a constitutive extended dimer

**Figure 1:**
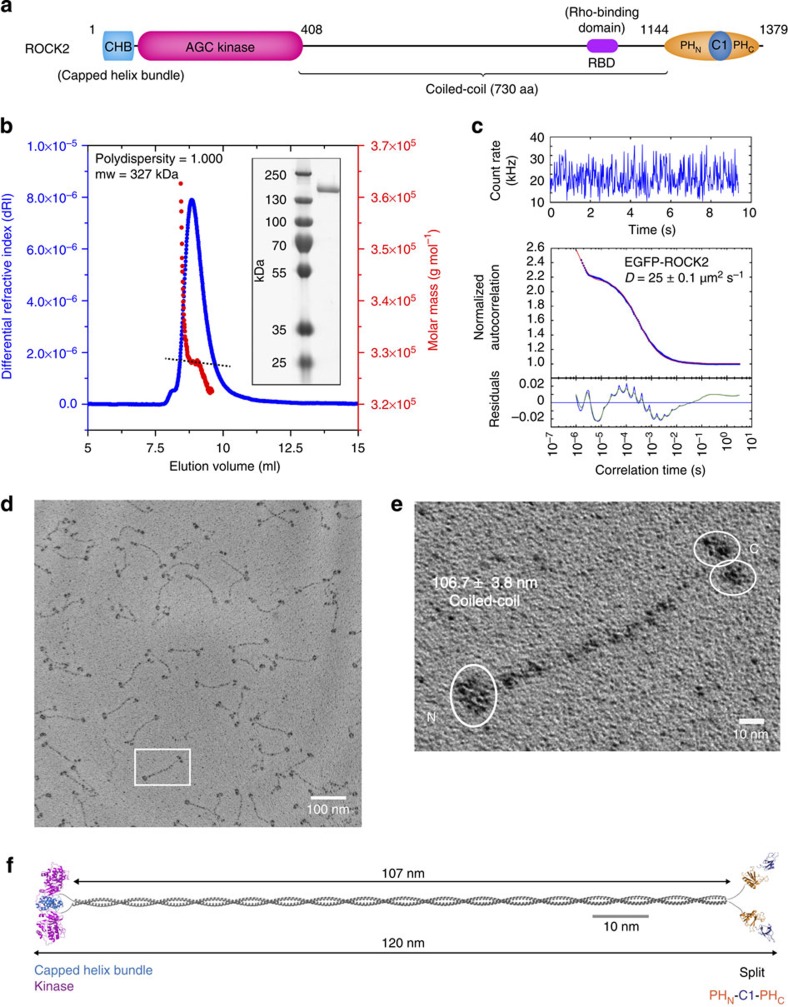
ROCK2 forms a semi-rigid extended coiled-coil dimer. (**a**) ROCK2 domain composition. N-terminal kinase and C-terminal membrane-binding PH and C1 domains are separated by 730 amino acids of predicted coiled-coil. (**b**) Static light-scattering analysis of recombinant ROCK2. ROCK2 has a molecular weight of 327 kDa and a polydispersity index of 1.000. (**c**) FCS of EGFP-labelled ROCK2. ROCK2 was isolated from mammalian cells using FSEC and its diffusion coefficient determined by FCS. The value of 25 μm^2^ s^−1^ is consistent with an extended, semi-rigid particle. (**d**) Rotary shadowing electron microscopy of ROCK2. Dimeric ROCK2 particles consist of globular N- and C-terminal domains separated by a long coiled-coil. (**e**) Zoomed image of a representative particle. The particles show a clear asymmetry between their ends: while one end appears more compact, the other end is characterized by two regions of electron density. The length of the coiled-coil is 106.7±3.8 nm (*n*=10). (**f**) Scale model of ROCK2. The N-terminal kinase domains dimerize via their capped helix bundle domains (PDB: 2F2U). A parallel coiled-coil of 107 nm joins the kinase domains to the monomeric C-terminal membrane-binding regulatory domains (PDBs: 2ROV (C1), 2ROW (split PH)), giving a maximum particle length of 120 nm. We note that the scale model gives the impression of a longer particle than the rotary-shadowed images of ROCK2, but that this is a consequence of the shadowing of the globular domains, which gives the impression of domains that are larger than they actually are.

To probe the structure and conformation of a full-length Rho kinase, we expressed a gene-optimized construct of human ROCK2 in baculovirus-infected insect cells. Mass spectrometry revealed the presence of mono- and diphosphorylated species (Supplementary Fig. 1A–E) corresponding to two adjacent serines (1,352–1,353) in its C terminus. The protein was confirmed to be a single dimeric species with a mass of 327 kDa and a root mean square radius of 12 nm by static light scattering (Fig. 1b), indicating a highly extended conformation. We confirmed that ROCK2 adopts an extended conformation in mammalian cells by measuring its diffusion coefficient using fluorescence correlation spectroscopy (FCS; Fig. 1c and Supplementary Fig. 2A–D). The low-resolution structure of purified ROCK2 was determined using rotary shadowing electron microscopy and revealed an extended particle, 120 nm in length with globular domains at both ends (Fig. 1d,e). The kinase and membrane-binding domains are separated by a rod of coiled-coil, 106.7±3.8 nm, which corresponds to the length of a canonical coiled-coil of 730 amino acids (1.46 Å rise per amino acid). The particles display a clear asymmetry in the arrangement of their globular domains, with one end of the particle adopting a more compact assembly and the other end displaying what appear to be two individual domains (Fig. 1e). Our observations are therefore consistent with a parallel dimer mediated by a canonical coiled-coil, confirming previously published high-resolution structures of fragments of the coiled-coil^14^,^15^,^16^,^17^. Comparison of the measured diffusion coefficient of 25–27 μm^2^ s^−1^ with that of a stiff rod of the dimensions observed for the recombinant protein (18 μm^2^ s^−1^) indicates that ROCK2 also adopts an extended, semi-rigid, conformation in cells (Supplementary Fig. 2D). We constructed a scale model of the full-length protein by joining the high-resolution structures of the dimeric catalytic domain and the monomeric membrane-binding domains with a coiled-coil of 107 nm (Fig. 1f).

### Active ROCK2 signalling depends on the length of its coiled-coil

The extended structure of ROCK2 immediately suggested that its activity might not be autoinhibited by its regulatory domains. It struck us that, despite low sequence conservation in the coiled-coil of ROCK2, its length is remarkably well conserved across more than 600 million years of evolution (Supplementary Fig. 3A,B). We wondered whether the length of the coiled-coil determines the correct positioning of the kinase domains of ROCK with respect to the membrane and, thereby, the localization of its catalytic activity together with its substrates in the cortical actin cytoskeleton. To investigate this, we evaluated a series of ROCK2 truncation mutants inside cells. Constructs were designed to shorten the coiled-coil while preserving its register and the catalytic activity of the kinase domains. To avoid heterodimerization of ROCK2 truncation mutants with endogenous ROCK2, and therefore incorrect assembly of both proteins, we also made a CRISPR/Cas9 knockout^27^ of ROCK2 in NIH3T3 fibroblasts (Supplementary Fig. 3C). The ROCK2 knockout cells showed no obvious phenotypic defect in the morphology of their actin cytoskeleton when compared with wild-type NIH3T3 cells (Supplementary Fig. 3D), indicating that ROCK1 can compensate for ROCK2.

**Figure 2:**
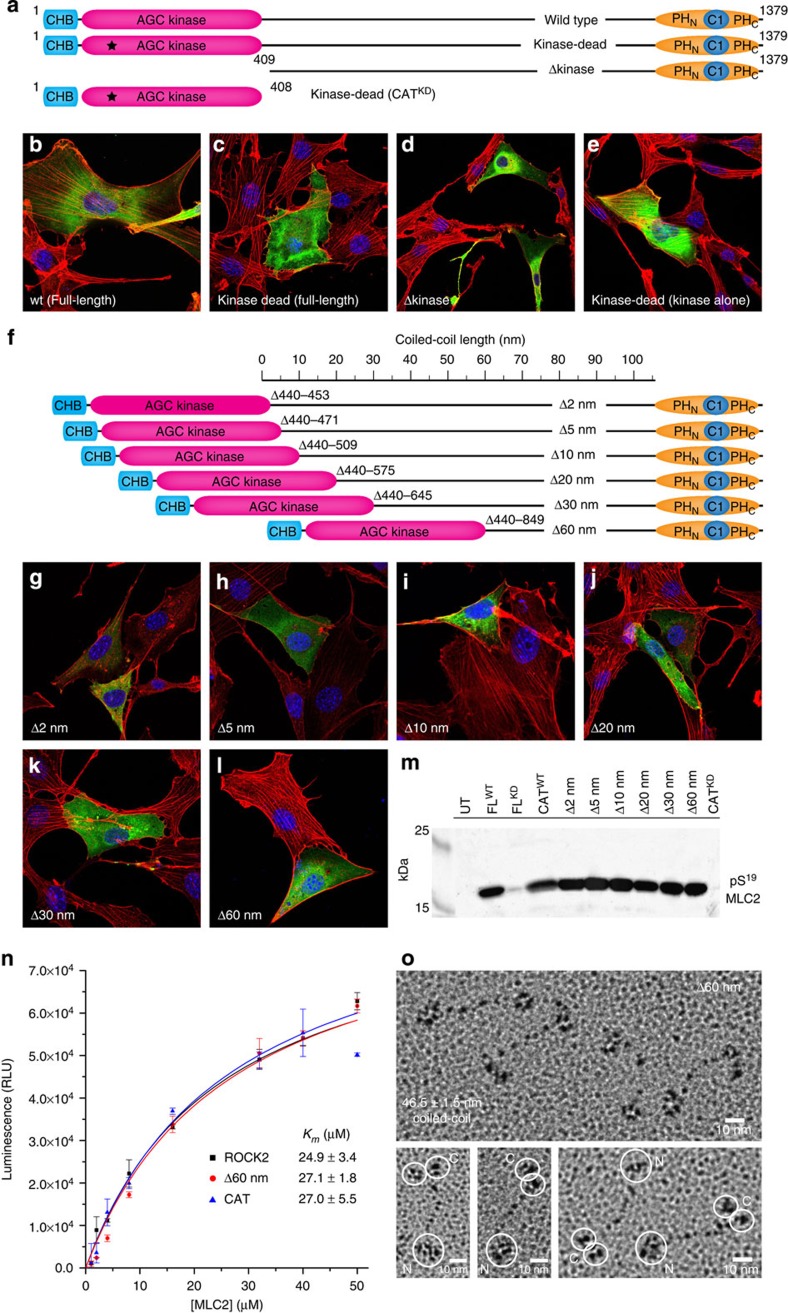
ROCK2 coiled-coil length regulates activity *in vivo* but not *in vitro*. (**a**) Schematic illustrating ROCK2 constructs used in **b**–**e**. (**b**) NIH3T3 ΔROCK2 cells expressing ROCK2^WT^ have stress fibres indistinguishable from untransfected cells. (**c**) ROCK2^K112A^ (kinase-dead)-expressing cells exhibit a dominant-negative phenotype characterized by the complete loss of stress fibres, while untransfected cells show normal stress fibres. (**d**) Cells expressing ROCK2^409–1,379^, a construct comprising the entire coiled-coil and the membrane-binding C1 and PH domains, also exhibit the complete loss of stress fibres. (**e**) Cells overexpressing ROCK2^1–409 K112A^, a kinase-dead construct of the catalytic domain alone, exhibit normal stress fibres. (**f**) Schematic illustrating truncation constructs of ROCK2 used in **g**–**l**. (**g**) NIH3T3 ΔROCK2 fibroblasts expressing ROCK2 truncated by just 2 nm show normal stress fibres. (**h**) NIH3T3 ΔROCK2 fibroblasts expressing ROCK2 truncated in its coiled-coil by 5–60 nm (**h**–**l**) exhibit a complete loss of stress fibres. (**m**) *In vitro* kinase assay of ROCK2 mutants. Immunoblot of pS^19^ MLC2 following incubation with EGFP-tagged ROCK2 immobilized in a GFP-Trap 96-well plate. ROCK2^WT^ is active against MLC2, while kinase-dead ROCK2^K112A^ is completely inactive. ROCK2^Δ2^, ROCK2^Δ5^, ROCK2^Δ10^, ROCK2^Δ20^, ROCK2^Δ30^ and ROCK2^Δ60^ are all active against MLC2, as is the isolated kinase domain (CAT). (**n**) *In vitro* kinase assay of ROCK2^wt^, ROCK2^Δ60^ and ROCK2^CAT^. All three constructs show the same specific activity against MLC2. Error bars are the s.d. of three independent measurements. (**o**) Rotary shadowed electron microscopy of ROCK2^Δ60^. ROCK2^Δ60^ is an extended particle with a coiled-coil length of 46.5±1.5 nm (*n*=10). Asymmetry is illustrated by four particles with a single region of electron density at one end of the particle and two clear regions of electron density at the other.

Ectopic expression of kinase-dead ROCK1 or ROCK2 leads to the dominant-negative loss of actin stress fibres in cells^28^,^29^. The same dominant-negative phenotype is obtained by overexpressing the regulatory PH–C1 domain of ROCK1 or ROCK2 (refs 7, 28, 29), indicating that the dominant-negative phenotype is driven by interference with the cellular functions of both endogenous ROCK1 and ROCK2. We confirmed this by expressing wild-type ROCK2 or kinase-dead ROCK2 in both wild-type NIH3T3 and ROCK2 knockout NIH3T3 fibroblasts and staining actin fibres with phalloidin (Fig. 2a–c; Supplementary Fig. 3F). The same loss of stress fibres was observed on expression of a construct lacking the kinase domain (Fig. 2a,d). To probe whether the dominant-negative phenotype was driven by the correct subcellular localization of the kinase, we expressed the kinase-dead catalytic domain alone (CAT^KD^). In contrast to kinase-dead full-length ROCK2, the inactive catalytic domain alone did not cause the loss of stress fibres, even at high expression levels (Fig. 2a,e), indicating that the correct subcellular localization of the kinase is critical for its function. This is consistent with findings in Madin–Darby canine kidney cells in which microinjection of a plasmid encoding the split PH domain inhibits stress fibre formation, but both the coiled-coil and the kinase-dead catalytic domains have no effect^7^.

Next, we investigated whether the length of the coiled-coil of ROCK2 might regulate the interaction of its catalytic domain with downstream substrates in cells. We transfected ΔROCK2 NIH3T3 cells with constructs truncated by 2, 5, 10, 20, 30 and 60 nm in the N-terminal region of the coiled-coil (Fig. 2f), which corresponds to the most sequence-divergent region of ROCK. For truncations of 5 nm or more, we observed a complete loss of stress fibres in cells expressing these constructs (Fig. 2h–l) that was indistinguishable from cells expressing kinase-dead ROCK2 (Fig. 2c). Truncating the coiled-coil by just 2 nm, or 31 amino acids, was insufficient to promote stress fibre loss (Fig. 2g), which is less than the s.d. of ±3.5 nm in predicted coiled-coil length among ROCK orthologues (Supplementary Fig. 3A,B). We observed robust activity for all truncation mutants, while kinase-dead ROCK2 was completely inactive in an *in vitro* kinase assay (Fig. 2m).

To confirm that these proteins were not just active but also correctly assembled and soluble, we purified them from NIH3T3 ΔROCK2 cell lysates by fluorescence size exclusion chromatography (FSEC). The truncated proteins eluted as single species with progressively longer retention times corresponding to shorter coiled-coils (Supplementary Fig. 3E, ROCK2 Δ60 nm), indicating that they are properly assembled. To confirm that truncation of the coiled-coil does not influence the specific activity of ROCK2, we purified the 60 nm truncation mutant from insect cells and found its activity to be indistinguishable from full-length ROCK2 (Fig. 2n). To rule out any influence of the regulatory domains on ROCK2 activity *in vitro*, we also assayed the activity of the recombinant catalytic domain (ROCK2^1–408^), which is constitutively dimeric in solution, consistent with previous reports^12^. We observed that the activity of the isolated kinase domain is identical to that of both the full-length and 60-nm-truncated ROCK2 (Fig. 2n), consistent with a lack of autoregulation. Finally, electron microscopy of the truncated protein shows it to be an extended particle with a coiled-coil length of 46.5±1.5 nm, consistent with shortening of the full-length protein (106.7±3.8 nm) by the designed 60 nm (Fig. 2o).

### ROCK2 activity is coiled-coil sequence independent

**Figure 3:**
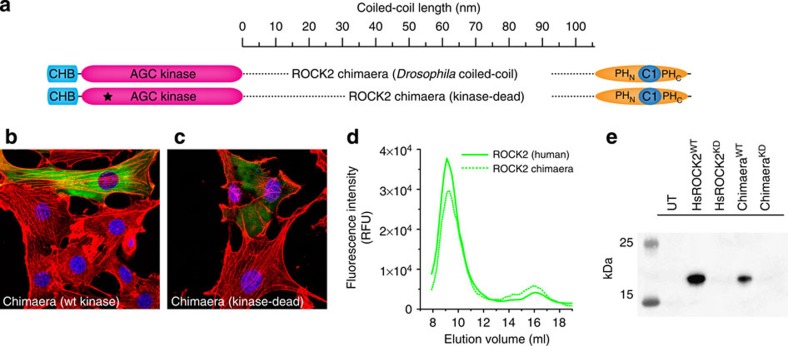
ROCK2 activity *in vivo* is coiled-coil sequence independent. (**a**) Schematic illustrating the construction of a ROCK2 chimera. The coiled-coil region of human ROCK2 (amino acids 414–1,141) was replaced with the corresponding region of *Drosophila melanogaster* (amino acids 418–1,133). A kinase-dead chimera contains the same inactivating mutation (K112A) as the kinase-dead human ROCK2. (**b**) NIH3T3 ΔROCK2 cells expressing the ROCK2 chimera show stress fibres indistinguishable from untransfected cells. (**c**) Expression of the kinase-dead chimera results in complete loss of stress fibres. (**d**) Fluorescence size exclusion chromatography of human ROCK2 and chimeric ROCK2 shows them to elute with identical retention volumes, indicating that the chimera is soluble and correctly folded. (**e**) *In vitro* kinase assay confirms that the ROCK chimera isolated from NIH3T3 ΔROCK2 cells is active against MLC2, while its kinase-dead counterpart is not.

To investigate whether the length of the coiled-coil, rather than its sequence, was the primary regulator of ROCK2 activity in cells, we replaced the coiled-coil with the coiled-coil of another protein. To ensure that the coiled-coil we chose was both inert in the cell and had similar mechanical properties to human ROCK2, we took advantage of the evolutionary sequence divergence in the ROCK coiled-coil itself. Noting that insects and humans shared a common ancestor ∼550 m years ago and that insects possess a single ROCK gene (Rok) compared with the two isoforms that all mammals have, we made a chimeric protein between the kinase and regulatory domains of human ROCK2 and the coiled-coil of *Drosophila melanogaster* Rok (Fig. 3a). While the kinase and regulatory domains of human ROCK2 and *Drosophila* Rok share 66% and 49% sequence identity, respectively, the coiled-coil is only 33% identical, of which the majority (∼28%) represent conserved hydrophobic residues that are essential for the structure and stability of the coiled-coil itself. We then asked the question whether this protein would exert a dominant-negative effect in cells (in the event that it was non-functional) or whether it would support normal stress fibre formation. Figure 3b shows that cells expressing the chimera exhibit normal stress fibres, while cells expressing a kinase-dead chimera are devoid of stress fibres (Fig. 3c), as is the case for kinase-dead human ROCK2 (Fig. 2c). We confirmed that the chimeric protein was properly folded by comparing its fluorescence size exclusion chromatography profile to that of human ROCK2 (Fig. 3d). We confirmed the activities of both the chimera and its kinase-dead counterpart with an *in vitro* kinase assay (Fig. 3e).

### ROCK2 activity is membrane and RhoA independent

**Figure 4:**
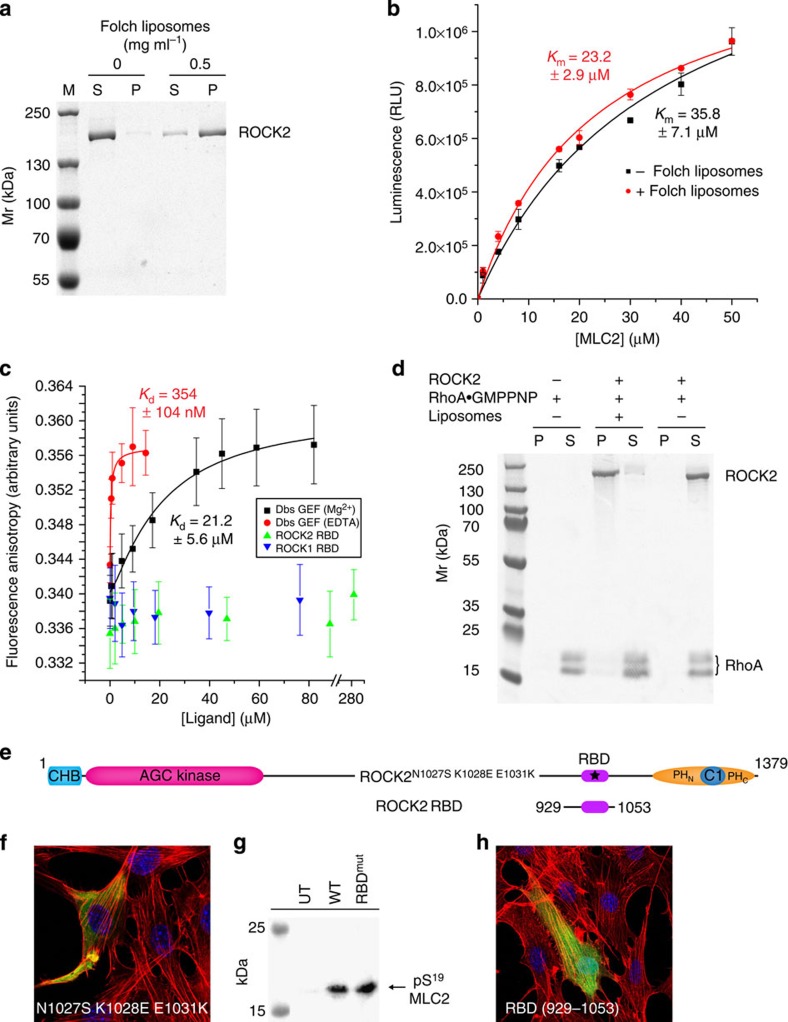
Membrane binding and kinase activity of ROCK2. (**a**) ROCK2 binds to membranes *in vitro*. Folch liposomes containing 0.5 mg ml^−1^ Folch brain lipids were sufficient to bind >90% of ROCK2. (**b**) Recombinant ROCK2 is active against the substrate MLC2 (black curve), but activity is not influenced by membrane binding (red curve). Error bars are the s.d. of three independent measurements. (**c**) Fluorescence anisotropy binding assay. ROCK1 and ROCK2 Rho-binding domains do not bind to RhoA·GMPPNP, even at very high protein concentrations. The Dbs exchange factor for RhoA binds to nucleotide-free RhoA (1 mM EDTA) with a binding constant of 354 nM, compared with a 60-fold weaker binding affinity for nucleotide-loaded RhoA (2 mM Mg^2+^). Error bars are the s.d. of 20 measurements. (**d**) RhoA does not bind to ROCK2 in the context of membranes. RhoA was incubated with ROCK2 bound to Folch liposomes in a liposome pelleting assay. RhoA is exclusively in the supernatant, while ROCK2 is exclusively in the pellet, indicating that membrane binding by ROCK2 does not facilitate its interaction with RhoA. (**e**) Schematic illustrating ROCK2 constructs used in **f**–**h**. (**f**) Cellular phenotype of overexpression of an RBD mutant of ROCK2. Immunostaining of ΔROCK2 NIH3T3 fibroblasts expressing ROCK2^N1027S K1028E E1031K^-EGFP shows normal stress fibres. (**g**) The RBD mutant has activity indistinguishable from ROCK2^WT^. (**h**) Cells overexpressing the RBD of ROCK2 (ROCK2^929–1,053^) also exhibit normal stress fibres.

Our model for ROCK2 (Fig. 1f) predicts that the intrinsic catalytic activity of the kinase would not be influenced by membrane binding or by RhoA. To test whether membrane binding influences the catalytic activity of ROCK2, we established conditions for the binding of ROCK2 to Folch liposomes (Fig. 4a) and assayed its activity. As Fig. 4b shows, membrane binding has no effect on its activity.

To test the effect of RhoA, we first sought to establish a complex between RhoA and ROCK2, noting that the reported binding site for RhoA is freely accessible in the full-length recombinant protein (Fig. 1a,e) and that the site is not modified (Supplementary Fig. 1D). Since RhoA binds its effector proteins in the GTP-bound state, we established a robust protocol for loading RhoA with the non-hydrolysable GTP analogue GMPPNP (see Methods). RhoA was confirmed to be >95% GMPPNP-loaded using ion-pairing reverse-phase high-performance liquid chromatography (HPLC; Supplementary Fig. 4A,B) and native mass spectrometry (Supplementary Fig. 4C). It was previously reported that the complex between RhoA and a fragment of ROCK1 was isolated using size exclusion chromatography^16^; therefore, to assay complex formation, we gel-filtrated a mixture of the two proteins at a 10:1 RhoA:ROCK2 stoichiometry. However, under physiological buffer conditions, we were unable to detect any interaction between the two proteins (Supplementary Fig. 4D) or the RBD fragments of ROCK1 and ROCK2 corresponding to the crystal structure (Supplementary Fig. 4E,F). To confirm that the lack of complex formation was not just a consequence of a low-affinity interaction, we measured the binding of the fragments to EGFP-RhoA.GMPPNP using fluorescence anisotropy. As a control, we measured the binding of the Dbs guanine nucleotide exchange factor to EGFP-RhoA.GDP and determined binding constants of 354±104 nM and 21.2±5.6 μM, in the presence of EDTA and Mg^2+^, respectively, which closely match published values for the complex between Dbs and the Rho GTPase Cdc42 (ref. 30). However, even at protein concentrations close to 300 μM, we were unable to detect any binding of either ROCK1 or ROCK2 to RhoA (Fig. 4c and Supplementary Fig. 4G). Finally, we assayed the ability of full-length ROCK2 to bind RhoA in the presence of Folch liposomes. Figure 4d shows that RhoA was found exclusively in the supernatant when incubated with ROCK2 bound to liposomes. To reconcile our findings with the original identification of the RBD of ROCK1 (ref. 18), we performed a yeast two-hybrid analysis of both ROCK1 and ROCK2 and the RBD fragment of ROCK1, noting that the full-length proteins were absent in the original study and that the experiments were only performed with the fusion partners in one direction. We observed a weak signal only between LexA-RhoA and GAD-ROCK1 RBD (Supplementary Fig. 4H, #3); however, this could not be reproduced when the LexA and Gal4 activation domains were switched (Supplementary Fig. 4H, #2). Significantly, we observed no interaction between full-length ROCK1 or ROCK2 and RhoA (Supplementary Fig. 4H, #9–12). Finally, despite observing no interaction between the two proteins by any method, we assayed the influence of RhoA on ROCK2 kinase activity *in vitro* using a 200-fold molar excess of GMPPNP-loaded RhoA. RhoA was not able to activate ROCK2 (Supplementary Fig. 4I); rather the presence of so much RhoA in the reaction suppressed activity by 35%, probably because of nonspecific interactions with the kinase and/or substrate. Taken together, our data show that RhoA does not bind to or activate either ROCK1 or ROCK2.

To confirm our findings in cells, we transfected ΔROCK2 NIH3T3 cells with a mutant of ROCK2 (Fig. 4e) designed to disrupt the salt bridges that stabilize the interface with RhoA that was observed in the crystal structure^16^. We were unable to detect any morphological defect in the actin cytoskeleton: transfected cells showed normal stress fibres that were indistinguishable from untransfected cells (Fig. 4f) and ROCK2 activity was unaffected by the mutations (Fig. 4g). Since it has also been suggested that the RBD of ROCK is essential for Rho-mediated stress fibre formation in 3T3 cells^26^, we asked the question whether overexpression of the RBD of ROCK2 in wild-type NIH3T3 cells would elicit a dominant-negative phenotype by titrating out RhoA. However, we again observed no defect in stress fibre formation (Fig. 4h).

### ROCK is not regulated by activation loop phosphorylation

**Figure 5:**
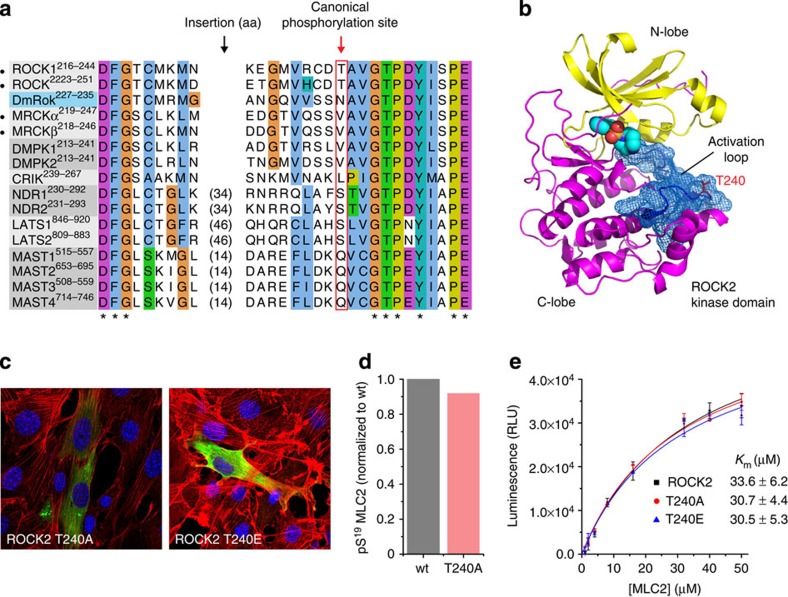
ROCK2 activity is independent of activation loop phosphorylation. (**a**) Alignment of activation loops for the ROCK subfamily of human AGC kinases. The canonical activation loop phosphorylation site of the AGC kinases is marked with a red box. The alignment includes a representative ROCK orthologue from the arthropod phylum (*D. melanogaster*), highlighted in blue. Proteins for which high-resolution structures exist of the unphosphorylated kinase domain with an ordered activation loop in the conformation characteristic of active eukaryotic protein kinases are indicated with a closed circle. (**b**) Active conformation of the ROCK2 kinase domain illustrated by the crystal structure (PDB ID: 2F2U). The ordered activation loop is highlighted in blue mesh, while the unphosphorylated canonical phosphorylation site (ROCK2 T240) is indicated in red. (**c**) Immunostaining of NIH3T3 ΔROCK2 cells expressing a non-phosphorylatable EGFP-ROCK2^T240A^ or a phosphomimetic EGFP-ROCK2^T240E^. Cells show normal stress fibres indistinguishable from untransfected cells. (**d**) ROCK2^T240A^ activity against MLC2 is indistinguishable from wild-type ROCK2 *in vitro*. EGFP-ROCK2 was immobilized in wells of a GFP-Trap 96-well plate and subjected to an *in vitro* kinase assay. Immunoblot against pS^19^ MLC2. ROCK2^T240A^ activity was normalized to ROCK2^WT^ using EGFP fluorescence. (**e**) *In vitro* kinase assay of ROCK2^WT^, ROCK2^T240A^ (non-phosphorylatable) and ROCK2^T240E^ (phosphomimetic). Error bars are the s.d. of three independent measurements. All three constructs show the same specific activity against MLC2.

Many eukaryotic kinases are regulated by phosphorylation of their activation loops and phosphorylation, although not observed to date, might regulate ROCK activity. However, evidence suggests that this is likely not the case. First of all, while the human Rho kinases contain a threonine in their activation loop at the position corresponding to that phosphorylated in many serine/threonine kinases of the AGC family, arthropod Rho kinases encode an asparagine. Furthermore, Rho kinases belong to a subfamily of the AGC kinases in which most members do not carry a phosphorylatable residue at the canonical position (Fig. 5a and Supplementary Fig. 5), yet they adopt active conformations^31^ in all structures determined to date^11^,^12^,^32^ (Fig. 5b) and recombinant ROCK2 is active against MLC2 *in vitro* in the absence of activation loop phosphorylation (Fig. 4b and Supplementary Fig. 1A–E). Nevertheless, human ROCK1 and ROCK2 possess a threonine at the canonical position, raising the question of whether this is relevant *in vivo*. To test whether mutation of T240 could impair/enhance stress fibre formation in cells, we transfected ΔROCK2 NIH3T3 fibroblasts with an activation loop mutant of ROCK2 in which T240 is mutated to either alanine (non-phosphorylatable) or glutamate (phosphomimetic). We observed identical morphology of the actin cytoskeleton in cells expressing ROCK2^T240A^ or ROCK2^T240E^ (Fig. 5c), both of which were indistinguishable from ROCK2^wt^-expressing cells (Fig. 2b and Supplementary Fig. 3F). These results are further supported by the observation that ROCK2^T240A^ is as active as ROCK2^wt^ when isolated from 3T3 fibroblasts (Fig. 5d), and the purified recombinant proteins exhibit the same activity as ROCK2^WT^ *in vitro* (Fig. 5e), indicating that activation loop phosphorylation likely does not regulate catalytic activity.

## Discussion

We have determined the low-resolution structure of a full-length ROCK and demonstrate that its kinase activity is not influenced by its regulatory domains, binding to membranes, RhoA, or by phosphorylation. We show here instead that the coiled-coil, which is highly conserved in length, but not sequence, plays a critical, and previously unappreciated role in ROCK signalling.

ROCK2 forms a constitutive, parallel, coiled-coil dimer in which a 107-nm coiled-coil links the membrane-binding regulatory domains to the kinase domains. The extended conformation of ROCK2 immediately appeared incompatible with autoinhibition of the kinase domains by the regulatory domains and/or the Rho-binding domain, which we confirmed by comparing the activity of the isolated catalytic domain with the full-length protein. Robust activity *in vitro* suggested that ROCK2 might be constitutively active. We reasoned that this might not present a problem to the cell if phosphotransfer by ROCK2 only occurred when substrate and kinase were correctly positioned in the cell. While ectopic expression of kinase-dead full-length ROCK2 promotes the dominant-negative phenotype of actin stress fibre loss, expression of the kinase-dead catalytic domain alone does not, indicating that subcellular localization of the kinase is of fundamental importance to downstream signalling. These findings are supported by previous observations that microinjection of antibodies against the PH domain block stress fibre formation with no apparent effect on ROCK2 activity. Expression of a regulatory domain construct of ROCK2 mutated in its PH domain also fails to elicit a dominant-negative effect on the actin cytoskeleton in cells^33^. Inspection of the structure of the PH domain^13^ reveals that the two mutated amino acids are buried in the hydrophobic core of the domain, indicating that the mutant PH domain was likely incorrectly folded. Our findings, in combination with those of others, point to displacement of endogenous ROCK1 or ROCK2 as the driver of the dominant-negative phenotype, and not *trans*-inhibition of the endogenous kinase by ectopically expressed regulatory domain constructs, as has previously been suggested^26^,^33^.

Since ROCK is localized to the plasma membrane by its membrane-binding domains and the length, but not the sequence of its coiled-coil is conserved, we speculated that the coiled-coil separating the kinase and membrane-binding domains might function as a molecular ruler. We found that truncation of the coiled-coil by just 5 nm was sufficient to abrogate stress fibre formation. We purified a 60-nm truncation mutant of ROCK2 and confirmed that it was both correctly folded and as active as the full length, wild-type kinase. We concluded that ROCK2 is constitutively active and that stress fibre formation is a function of kinase–substrate engagement in the cell. Our model is further supported by the observation that the length of the coiled-coil of ROCK is evolutionarily conserved throughout the animal kingdom (Supplementary Fig. 3A,B). We note that nematode worms (nematoda) and flatworms (platyzoa) have ROCK proteins with shorter coiled-coil regions, but that the predicted lengths of the coiled-coil also cluster together (Supplementary Fig. 3A). It is particularly interesting to note that the emergence of ROCK coincides with the evolution of multicellularity. The earliest record of ROCK can be found in the unicellular eukaryote *Capsaspora owczarzaki*, which also encodes a complete set of proteins involved in integrin-mediated cell adhesion and signalling^34^. By comparison, choanoflagellates and fungi, which do not possess a ROCK gene, have selectively lost various components of the integrin-signalling machinery. Given the role of ROCK in regulating actin dynamics at focal adhesions, it is tempting to speculate that the emergence of ROCK together with the integrin-mediated signalling machinery was a crucial event in metazoan evolution.

Our data show that neither membrane-binding nor RhoA influences the catalytic activity of the kinase, as is predicted by the extended structure of the ROCK2 dimer. Previous studies, however, suggested a direct interaction between ROCK and RhoA^16^,^18^,^28^,^35^,^36^. These studies relied on immobilization of one component on a solid support and overlaying the second component, yeast two hybrid analysis of fragments of the ROCK coiled-coil or indirect assays such as inhibition of nucleotide dissociation. However, residues in RhoA identified as important for effector binding do not always correspond to surfaces of interaction subsequently identified in structural studies. In the case of the structure of the RhoA:ROCK1 RBD complex, neither solution data nor mutagenic data were presented to validate the interface observed. In contrast, this is the first study that has addressed the direct interaction of RhoA with a full-length ROCK in solution. While we cannot exclude the possibility that technical reasons may have contributed to our inability to detect an interaction, a direct interaction in solution has never been quantified. It is also worth noting that binding of RhoA to the coiled-coil of ROCK 20 nm proximal to its membrane-binding domains, as is predicted by the structure of the complex, appears incompatible with membrane anchoring of RhoA.

Activation loop phosphorylation is one of the most common regulatory mechanisms of eukaryotic protein kinases. Many of the AGC kinases are notable for one or two additional regulatory phosphorylation sites in their C-terminal tails that are required for maximal catalytic activity^37^,^38^. The Rho and MRCK kinases, however, differ in two key respects. First, their activation loops are fully ordered and adopt active conformations in the absence of phosphorylation^11^,^12^,^31^,^32^, while residues that stabilize the phosphate group are not conserved in these kinases. The lack of requirement for activation loop phosphorylation has also been observed in the G-protein-coupled receptor kinases^39^, another branch of the AGC kinase family (Supplementary Fig. 5A). Second, the hydrophobic motif is sandwiched in a dimeric interface, mutation of which results in a monomeric kinase domain and a corresponding loss of activity^40^. We therefore judge that phosphorylation is highly unlikely based on the lack of accessibility of the motif. We show here that non-phosphorylatable and phosphomimetic activation loop mutants of human ROCK2 exhibit identical catalytic properties to the wild-type enzyme and that cells overexpressing either mutant are indistinguishable from wild-type ROCK2-expressing cells in the morphology of their actin cytoskeleton. We therefore conclude that, in contrast to the subfamily of AGC kinases regulated by both activation loop and hydrophobic motif phosphorylation, the evolutionarily older ROCK subfamily is likely not regulated by phosphorylation of either motif.

Other regulatory mechanisms that have been described for protein kinases include activation by accessory proteins and steric occlusion of the catalytic cleft^31^. The cyclin-dependent kinases, for example, are inactive in the absence of their cognate cyclin, which, in binding to the N-lobe of the kinase, reorients the αC helix for productive phosphotransfer^41^. The N-lobe of Cdk2 in complex with cyclin A superimposes on the N-lobe of ROCK2 with a r.m.s.d. of 0.98 Å over 66 Cα atoms (Supplementary Fig. 5B), and, critically, the conserved glutamate of the αC helix contacts the β3 lysine to create an ion pair essential for ATP-binding and catalysis (Supplementary Fig. 5C). The capped helix bundle domain of ROCK, in combination with the C-tail of the kinase domain, fulfills an equivalent role to cyclin A by binding to the N-lobe of the kinase domain and maintaining its active conformation. Indeed, ROCK1 is one of only a few kinases crystallized with its intact protein substrate, RhoE^42^. Comparison of the kinase domains of ROCK1 and ROCK2 in the presence and absence of substrate, respectively, shows them to be essentially identical (r.m.s.d.=0.96 Å).

Steric occlusion of the catalytic cleft is an inhibitory mechanism employed by various protein kinases in a variety of ways. Protein kinases A and C, for example, are maintained in catalytically competent, but inactive, conformations by engagement of a pseudosubstrate presented by a regulatory subunit or intramolecularly, respectively^43^,^44^. Focal adhesion kinase, by contrast, blocks access to its catalytic cleft with its regulatory domains^45^. However, our structural and biochemical findings do not support the autoinhibition of ROCK activity by its regulatory domains. While we cannot exclude that cellular inhibitors of ROCK may exist, no such inhibitors have so far been described.

**Figure 6:**
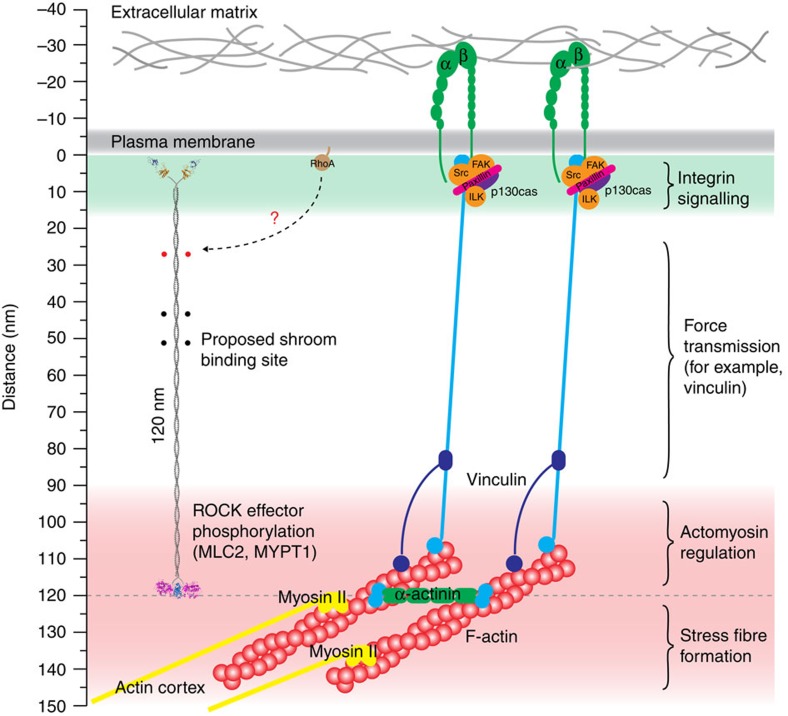
A molecular ruler governs ROCK access to substrates. ROCK-mediated actomyosin contraction. Functional model of ROCK with respect to the plasma membrane, focal adhesions and components of the actomyosin contractile machinery, including ROCK substrates, found in the actin cortex. All components are drawn approximately to scale. ROCK binds to the plasma membrane via its ‘split' PH and C1 domains, although the precise nature of the membrane ligands is as yet unknown. The coiled-coil of ROCK bridges the membrane to substrates located in the actin cortex at a distance of 120 nm. Scaffold proteins, such as Shroom, may serve to stabilize the orientation and/or flexibility of the coiled-coil, thereby restricting the conformational space sampled by the catalytic domains. Short conserved segments of the coiled-coil (indicated with closed circles) may serve as interaction surfaces for scaffold proteins. The link between RhoA, ROCK and downstream actomyosin contraction remains to be determined. We hypothesize that force transducers, such as talin and vinculin, regulate the positioning of the cytoskeletal machinery, and thus ROCK substrates such as MLC2 and MYPT1, with respect to focal adhesions and the plasma membrane.

Taken together, our data support the following model (Fig. 6). ROCK is a constitutively active kinase localized to the plasma membrane via its C1 and PH domains, the ligands of which are still unknown. Scaffolding proteins may serve to organize ROCK with respect to its substrates by refining (and perhaps limiting) the orientation of its coiled-coil. Within this framework, ROCK is positioned for substrate engagement by bridging a fixed distance between the membrane and its substrate. ROCK proteins with shorter coiled-coil spacers are unable to engage substrates, and despite being active, lead to cytoskeletal defects. Perhaps, the most prominent scaffold protein that has been described as a ROCK interaction partner is Shroom. The Shroom (Shrm) proteins play essential roles in tissue development by mediating the formation of contractile actomyosin networks that facilitate changes in cell shape^46^. Containing both actin- and ROCK-binding domains^47^,^48^, Shrm may serve to scaffold ROCK substrates in the cortical actomyosin network together with ROCK itself.

While many substrates have been proposed for ROCK, many remain to be validated as direct substrates. However, our model implies that such substrates are most likely dimeric and localized to the actin cortex at a distance of ∼120 nm beneath the plasma membrane. A recent biophysical attempt to measure the distance between the plasma membrane and the actin cortex arrived at a figure of 128±28 nm (ref. 49), a distance that would place the kinase domains of ROCK2 in the vicinity of its substrates. Super-resolution iPALM microscopy of integrin-based focal adhesions also places actin in a layer 50–150 nm from the plasma membrane^50^. Known ROCK substrates include MLC2, monomeric by itself, but dimeric in complex with the myosin heavy chain, and the myosin phosphatase targeting subunit MYPT1, itself a dimer.

The question remains, however, under what conditions do substrate and enzyme engage? One possibility is that cytoskeletal tension controls the positioning of both kinase and substrate. As such, the focal adhesion proteins talin and vinculin have been proposed to regulate force transmission between integrins and actin^51^,^52^,^53^. Talin itself has been shown to adopt a polarized conformation in the focal adhesion complex where it bridges the cytoplasmic tails of integrins to actin^50^. Rok depletion in *Drosophila* is associated with decreased tension and reduced actin accumulation^54^, while increased cytoskeletal tension correlates with tumour progression^55^, consistent with the upregulation of ROCK that has been observed in metastatic cancer cells^56^. Whilst ROCK clearly modulates cytoskeletal tension through phosphorylation of RMLC, it is also clear that cytoskeletal tension has the potential to regulate ROCK in a reciprocal manner by modulating the spatial positioning of its substrates with respect to the membrane.

Our findings indicate that the activity of the Rho kinases is regulated by the spatial positioning of kinase and substrate in the cell, much like the clutch in a car engine determines whether the car is in gear or not. The engine, or kinase, is always running, but the car, or cell, doesn't move unless the clutch, or substrate, is engaged. This represents a new paradigm in kinase–substrate regulation, whereby substrate specificity and corresponding activity are simply governed by the precise spatial positioning of enzyme and substrate.

## Methods

### ROCK2 truncation constructs

Human ROCK2 coiled-coil truncation constructs were generated using strand overlap extension PCR and are described by the deletion of the following amino-acid segments: Δ440–453 (Δ2 nm), Δ440–471 (Δ5 nm), Δ440–509 (Δ10 nm), Δ440–575 (Δ20 nm), Δ440–645 (Δ30 nm) and Δ440–849 (Δ60 nm). The human ROCK2—*Drosophila* Rok chimera was constructed by splicing together human ROCK2 1–413 (kinase domain), residues 418–1,133 of *Drosophila* Rok (coiled-coil) and residues 1,142–1,379 (regulatory domains) of human ROCK2.

### Protein purification

Human ROCK2, ROCK2^T240A^, ROCK2^T240E^, ROCK2^Δ440–849^ (Δ60 nm) and ROCK2^1–408^ (kinase domain alone) were expressed in Sf9 insect cells from a synthetic gene (GenScript) using the Bac-to-Bac system (Invitrogen) with a N-terminal His_10_-StrepII-tag. Cells were lysed in 50 mM Tris pH 7.5, 140 mM KCl, 10 mM NaCl, 1 mM tris-(2-carboxyethyl)phosphine (TCEP), 2 mM benzamidine, 1 mM phenylmethylsulphonyl fluoride, 100 μM bestatin, 14 μM E-64, 10 μM pepstatin and 1 μM phosphoramidon. ROCK2 was purified using NiNTA affinity purification and the tag cleaved overnight at 4 °C with TEV protease. ROCK2 was then purified using anion exchange chromatography in 50 mM Tris pH 8.0, 1 mM TCEP, 0–1 M NaCl). Peak fractions were pooled, concentrated, gel-filtrated into storage buffer (50 mM Tris pH 7.5, 1 mM TCEP, 140 mM KCl and 5% glycerol) and frozen at −80 °C.

GST-RhoA^1–181^ was purified using glutathione sepharose affinity purification in 50 mM Tris, pH 7.4, 150 mM KCl, 2 mM MgCl_2_ and 1 mM TCEP. RhoA^1–181^ was cleaved from the beads overnight using TEV protease. The protein was concentrated and applied to a Superdex 75 10/300 gel filtration column equilibrated in the same buffer. Peak fractions were concentrated and stored at −80 °C.

The guanine exchange factor Dbs^622–965^ was expressed in *E. coli* BL21 (DE3) with a N-terminal His_6_-tag. Dbs^622-965^ was purified using NiNTA affinity chromatography in 50 mM phosphate pH 7.5, 150 mM NaCl. Peak fractions were pooled, concentrated and applied to a Superdex 75 gel filtration column equilibrated in 50 mM Tris pH 8.0, 1 mM TCEP, 5% Glycerol and 200 mM NaCl. Peak fractions were pooled, concentrated to 3 mg ml^−l^ and stored at −80 °C.

MLC2 (MYL12B) was expressed in *E. coli* BL21 DE3 cells as a N-terminal GST-fusion protein. MLC2 was purified with affinity chromatography using glutathione sepharose beads in 50 mM Tris pH 7.5, 150 mM NaCl and 1 mM TCEP. Beads were washed and GST-MLC2 was cleaved with TEV overnight at 4 °C. MLC2 was concentrated and applied to a Superdex 75 gel filtration column equilibrated in 20 mM Tris pH 7.5, 150 mM NaCl and 1 mM TCEP. Peak fractions were pooled and concentrated to 3 mg ml^−1^ and stored at −80 °C.

The RBD fragments of human ROCK1 (947–1,015) and human ROCK2 (977–1,047) were expressed in *E. coli* BL21 (DE3) with a N-terminal His_6_-tag. Proteins were purified using NiNTA affinity chromatography in 50 mM Tris pH 7.5, 100 mM NaCl and 1 mM TCEP. Peak fractions were pooled, concentrated and applied to a Superdex 75 gel filtration column equilibrated in 20 mM Tris pH 7.5, 100 mM NaCl, 1 mM TCEP and 4 mM MgCl_2_. Both RBD fragments eluted as single peaks corresponding to a dimer of each fragment. Peak fractions were pooled and concentrated to 2.2 mg ml^−1^ (ROCK1 RBD) or 8.8 mg ml^−1^ (ROCK2 RBD) and stored at −80 °C.

### Fluorescence size exclusion chromatography

Cos7 or NIH3T3 cells were transiently transfected using Lipofectamine 2000 (Invitrogen) and 2 μg of DNA per well of a six-well plate. Twenty-four to seventy-two hours after transfection cells were harvested. Cells were lysed by three freeze-thaw cycles in 20 mM Tris pH 7.4, 140 mM KCl and 1 mM TCEP containing protease inhibitor cocktail (P8849, Sigma). Insoluble material was removed using ultracentrifugation at 124,000*g* for 15 min. The soluble fraction was loaded on a Superdex 200 size exclusion column equilibrated in lysis buffer. The fluorescence chromatogram was reconstructed by fractionating into a 96-well plate that could be read on a TECAN Infinite F500 fluorescence plate reader.

### Fluorescence correlation spectroscopy

FSEC peak fractions were analysed by FCS using a Confocor spectrofluorimeter (Carl Zeiss-Evotec, Jena, Germany) equipped with a 450-nm Argon laser (LASOS Lasertechnik GmbH, Jena, Germany) and a long-pass dichroic beam splitter (510 nm). Photons were recorded on an avalanche photodiode detector (SPCM-CD 3017), and fluorescence intensities were autocorrelated with a hardware correlator (ALV 5000, ALV, Langen, Germany). Data were analysed with the FCS ACCESS software (Carl Zeiss-Evotec). For every sample 10 consecutive measurements were recorded, each lasting a minimum of 10 s. The diffusion coefficient of a stiff rod with the dimensions of the recombinant protein was calculated using the relationship:


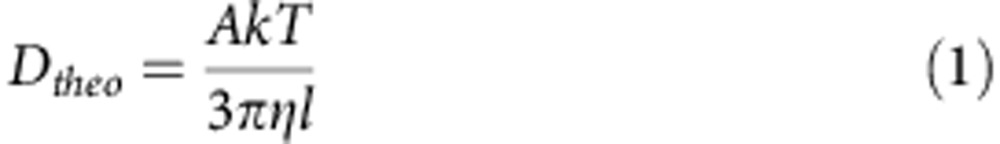


where


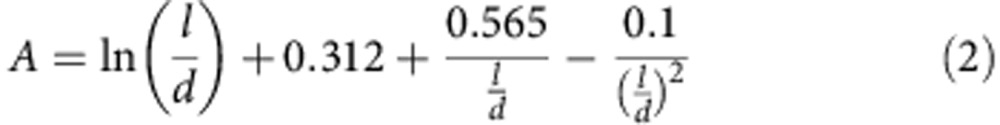


and *l*=length and *d*=diameter of the rod. Apparent molecular brightness was determined by dividing the average recorded fluorescence intensity of the time tracks by the number of particles in the confocal volume.

### Electron microscopy

Samples were diluted to a final concentration of 50 μg ml^−1^ in spraying buffer containing 100 mM ammonium acetate and 30% (v/v) glycerol, pH 7.4.

After dilution, the samples were sprayed on freshly cleaved mica chips and immediately transferred into a BAL-TEC MED020 high-vacuum evaporator equipped with electron guns. While rotating, the samples were coated with 0.6-nm platinum at an angle of 4–5°, followed by 10-nm carbon at 90°. The obtained replicas were floated off from the mica chips, picked up on 400 mesh Cu/Pd grids and inspected in an FEI T20 G2 transmission electron microscope operated at 80 kV. Electron micrographs were acquired using an FEI Eagle 4k charge-coupled device camera.

### Rhodamine-labelled sucrose-loaded vesicles (SLVs)

Folch fraction I lipids together with 6 μM Rhodamine-dihexadecanoyl-sn-glycero-3-phosphoethanaloamine (DHPE) were pipetted into borosilicate test tubes. Lipids were dried to a thin film using a dry nitrogen stream. One-millilitre 20 mM HEPES, pH 7.5, 0.3 M sucrose was added and the vesicles were vortexed vigorously. Vesicles were subjected to four freeze-thaw cycles in liquid nitrogen, followed by thawing in a sonicating water bath. SLVs were diluted in 20 mM HEPES, pH 7.5, 100 mM KCl and pelleted at 17,830*g* at room temperature (RT), 30 min. The vesicles were resuspended in 20 mM HEPES, pH 7.5, 100 mM KCl.

### Membrane-binding assay

For liposome-pelleting assays, 1 μM ROCK2 and/or 8 μM RhoA-GMPPNP were incubated together with or without 0.5 mg ml^−1^ Rhodamine-labelled sucrose-loaded vesicles (see above) at RT, 30 min. Samples were pelleted at 17,830*g*, 20 min at RT, loaded to SDS–PAGE and Coomassie-stained.

### RhoA loading with GMPPNP

RhoA (co-purified with GDP) was incubated with Dbs exchange factor (5:1) in a 100-fold molar excess of GMPPNP, 30 min at RT. RhoA was separated from Dbs and free nucleotide using size exclusion chromatography. The extent of GMPPNP loading was determined with ion-pairing reverse-phase HPLC and mass spectrometry (see below).

### Ion-pairing reverse phase HPLC

Ion-pairing reverse phase chromatography was performed using a Phenomenex C18 column (150 × 4.60 mm, 5 μm) with a EC 4/3 UNIVERSAL RP guard column (Macherey-Nagel). Runs were performed in isocratic mode (100 mM KH_2_PO_4_/K_2_HPO_4_ pH 6.5, 10 mM tetra-*n*-butylammonium bromide, 4.5 % CH_3_CN) at a flow rate of 1 ml min^−1^. Nucleotide elution volumes were monitored with ultraviolet absorbance at 260 and 280 nm.

### Intact mass spectrometry of ROCK2

Recombinant ROCK2 was injected on a C4 reverse phase column and eluted with a 12-min gradient from 3 to 98% acetonitrile. The column was coupled via an electrospray source to a Bruker Impact II Q-TOF instrument for mass determination.

### Native mass spectrometry of RhoA

The sample was transferred to a borosilicate emitter tip and directly infused into an LTQ-Orbitrap XL mass spectrometer (Thermo) using a source voltage of 1.5 kV. Spectra were recorded at a resolution of 60.000 at *m/z* 400 with 10 microscans at an AGC target value of 1E6, applying an in-source fragmentation of 80 kV. The averaged raw spectra of the +9 and +10 charge states were manually deconvolved using mMass^57^, before calculating the average masses for all peaks observed. The charge states +7 and +8 were omitted because of strong adduct signals that hampered deconvolution and analysis.

### Tryptic digest mass spectrometry of ROCK2

*Trypsin digestion and peptide identification via LC-MS/MS of ROCK2*. The protein was denatured in 2 M urea, reduced with 10 mM dithiothreitol (DTT), alkylated with 20 mM iodoacetamide and digested overnight using mass-spec-grade trypsin (Promega) at 37 °C. The digestion was stopped with trifluoroacetic acid and the peptides were desalted using custom-made C18 stagetips^58^. The peptides were separated on an Ultimate 3000 RSLC nano-flow chromatography system (Thermo-Fisher), using a pre-column for sample loading (PepMapAcclaim C18, 2 cm × 0.1 mm, 5 μm, Dionex-Thermo-Fisher) and a C18 analytical column (PepMapAcclaim C18, 50 cm × 0.75 mm, 2 μm, Dionex-Thermo-Fisher), applying a linear gradient from 2 to 30% solvent B (80% acetonitrile, 0.1% formic acid; solvent A 0.1% formic acid) at a flow rate of 230 nl min^−1^ over 60 min. Eluting peptides were analysed on a Q Exactive Plus Orbitrap mass spectrometer (Thermo-Fisher), equipped with a Proxeon nanospray source (Thermo Fisher), operated in a data-dependent mode. Survey scans were obtained in a mass range of 380–1,650 *m/z* with lock mass on, at a resolution of 70.000 at 200 *m/z* and an AGC target value of 3E6. The 12 most intense ions were selected with an isolation width of 2 Da, fragmented in the HCD cell at 27% collision energy and the spectra recorded at a target value of 1E5 and a resolution of 17,500. Peptides with a charge of +1 were excluded from fragmentation, the peptide match and exclude isotope features were enabled and selected precursors were dynamically excluded from repeated sampling for 10 s. Raw data were processed using the MaxQuant software package (version 1.3.0.5, http://www.maxquant.org/)^59^ and searched against a custom database containing the ROCK2 target sequence, as well as all *Spondoptera* spp. protein sequences available in Uniprot and the *Bombyx mori* reference proteome (www.uniprot.org). The search was performed with full trypsin specificity and a maximum of two missed cleavages at a protein and peptide false discovery rate of 1%. Carbamidomethylation of cysteine residues was set as fixed, oxidation of methionine, N-terminal acetylation and phosphorylation of serine, threonine and tyrosine as variable modifications—all other parameters were set to default. To cover potential unusual modifications we additionally performed a manual error-tolerant search for ROCK2 using MSconvert (http://proteowizard.sourceforge.net) with Mascot (version 2.2.0.7; www.matrixscience.com) as a search engine.

### Kinase assay

Kinase assays were performed using the ADP-Glo kit (Promega) according to the manufacturer's instructions. Kinase reactions contained 100 nM ROCK2, purified MLC2 substrate and 1 mM ATP in 20 mM Tris pH 7.5, 100 mM KCl, 1 mM TCEP, 0.05 mg/ml BSA, 2 mM MgCl_2_ and were incubated at RT, 40 min. Luminescence was measured using a TECAN Infinite F500 plate reader.

### Cell culture and confocal fluorescence microscopy

COS7, NIH3T3 and NIH3T3 ΔROCK2 fibroblasts were transfected with plasmids expressing C-terminal EGFP fusions of ROCK2. COS7 and NIH3T3 cells were obtained from the laboratory of F. Propst (MFPL). Cells for microscopic analysis were fixed with 2% formaldehyde and stained with AlexaFluor 594-phalloidin (Invitrogen, 1:100) and 4,6-diamidino-2-phenylindole (1 μg ml^−l^). Images were acquired on a Zeiss LSM710 confocal microscope equipped with 405, 488 and 561 nm lasers.

### CRISPR/Cas9 ROCK2 knockout NIH3T3 fibroblasts

Guide RNAs (gRNAs; 20 bp) were designed against the mouse ROCK2 gene using the CRISPR DESIGN tool from the Zhang laboratyory, MIT. gRNAs were subcloned into the lenti-CRISPR_v2 vector^60^. These constructs were packaged into lentivirus-like particles as previously described^61^. Briefly, HEK293T cells were transfected with 455 ng of GAG-POL packaging plasmid, 455 ng lentiCRISPR_v2 and 90 ng VSV-G envelope plasmid in six-well clusters using 3 μg polyethylenimine per 1 μg of plasmid DNA. Seventy-two hours post transfection, supernatants were harvested, filtered through a 0.45-μm filter and used to transduce NIH3T3 cells in the presence of 3 μg ml^−1^ polybrene. Seventy-two hours post transduction, cells were selected for a total of 1 week in the presence of 3 μg ml^−1^ puromycin (Invivogen). Surviving polyclonal cell pools were evaluated for ROCK2 expression by immunoblot and those with undetectable ROCK2 protein were used for further experiments. LC3 gRNA sequences: 5′-CACCGctggatgcaatacactccat-3′ and 5′-AAACatggagtgtattgcatccagC-3′.

### Fluorescence anisotropy

100 nM EGFP-RhoA in 20 mM Tris, pH 7.4, 150 mM KCl, 1 mM TCEP and either 2 mM MgCl_2_ or 1 mM EDTA was titrated with increasing concentrations of Dbs GEF, ROCK1 947–1,015 or ROCK2 977–1,047. Fluorescence anisotropy was measured in a Perkin Elmer LS50B fluorimeter (*λ*_ex_=488 nm, *λ*_em_=507 nm) at 25 °C. Ten measurements were made for each data point with an integration time of 10 s.

### *In vitro* kinase assay of ectopically expressed ROCK2

NIH3T3 ΔROCK2 fibroblasts expressing ROCK2-EGFP constructs were resuspended in lysis buffer-containing protease inhibitor cocktail (Sigma, P8849), freeze-thawed three times, clarified and the soluble fractions aliquoted into a 96-well GFP-multiTrap plate (Chromotek), 2 h at 4 °C. Wells were washed three times with lysis buffer and the fluorescence measured in a TECAN fluorescence plate reader. A master mix containing 20 μM purified MLC2 in 50 mM Tris pH 7.5, 140 mM KCl, 1 mM TCEP, 5 mM MgCl_2_ and 1 mM ATP was added to each well and incubated at RT, 1.5 h. Kinase reactions were terminated by adding SDS–PAGE loading buffer. Samples (320 ng MLC2) were assayed for phospho-MLC2 (Ser19; Cell Signaling Technology) by immunoblot.

### Yeast two hybrid

Plasmids were transformed into yCK580 yeast. Yeast was taken from plate and resuspended in 85-μl One-step-buffer (0.24 M LiAc, 47% PEG 3350, dH_2_O) with 10 μl 1 M DTT and 5 μl single-strand DNA (carrier sperm DNA). The mix was vortexed and 1–5 μl plasmid DNA added. After incubation for 30 min at 45 °C, samples were plated on −Leu, −Trp, ++Ade plates. Plates were grown at 30 °C for 48 h. To analyse protein–protein interactions, transformants were probed in a β-galactosidase liquid assay. Transformant colonies were grown overnight in 3 ml SD −Leu, −Trp, ++Ade medium, diluted in the morning to an optical density (OD) of 0.5 in SD −Leu, −Trp, ++Ade medium and grown to an OD of 1.8–2.0 (log phase). One-millilitre culture was taken at this point for TCA precipitation to check protein expression levels by immunoblot. OD (2.5; 1 OD=1 ml of a OD 1.0 culture) of each transformant was pelleted for 4 min, 3,000*g* at RT. The pellets were resuspended in 30 μl PBS and transferred into a flat transparent bottom 96-well plate. X-Gal solution (100 μl; 7.15 μM ß-Me, 0.1% SDS, PBS pH 7.5, 0.05% X-Gal) was added to each well to start the liquid assay. Plates were protected from light and scanned at different time points.

### Size exclusion chromatography of RhoA:ROCK complexes

Complex formation was assayed with the RBD fragments of ROCK1 and ROCK2 or with full-length ROCK2 and a 10-fold molar excess of RhoA-GMPPNP. Complex formation was followed by size exclusion chromatography on a Superose 6 gel filtration column equilibrated in 20 mM Tris pH 7.5, 1 mM TCEP, 4 mM MgCl_2_, with or without 100 mM NaCl.

## Additional information

**How to cite this article:** Truebestein, L. *et al.* A molecular ruler regulates cytoskeletal remodelling by the Rho kinases. *Nat. Commun.* 6:10029 doi: 10.1038/ncomms10029 (2015).

## Supplementary Material

Supplementary Information Supplementary Figures 1-5
