# Supplementary material for: A molecular ruler regulates cytoskeletal remodelling by the Rho kinases
Source: Nat Commun. 2015 Dec 1;6:10029. doi: 10.1038/ncomms10029 (PMC4686654; doi:10.1038/ncomms10029)
Supplement: Supplementary Information — Supplementary Figures 1-5 [file ncomms10029-s1.pdf]

Supplementary Figure 1. Mass spectrometry analysis of ROCK2.

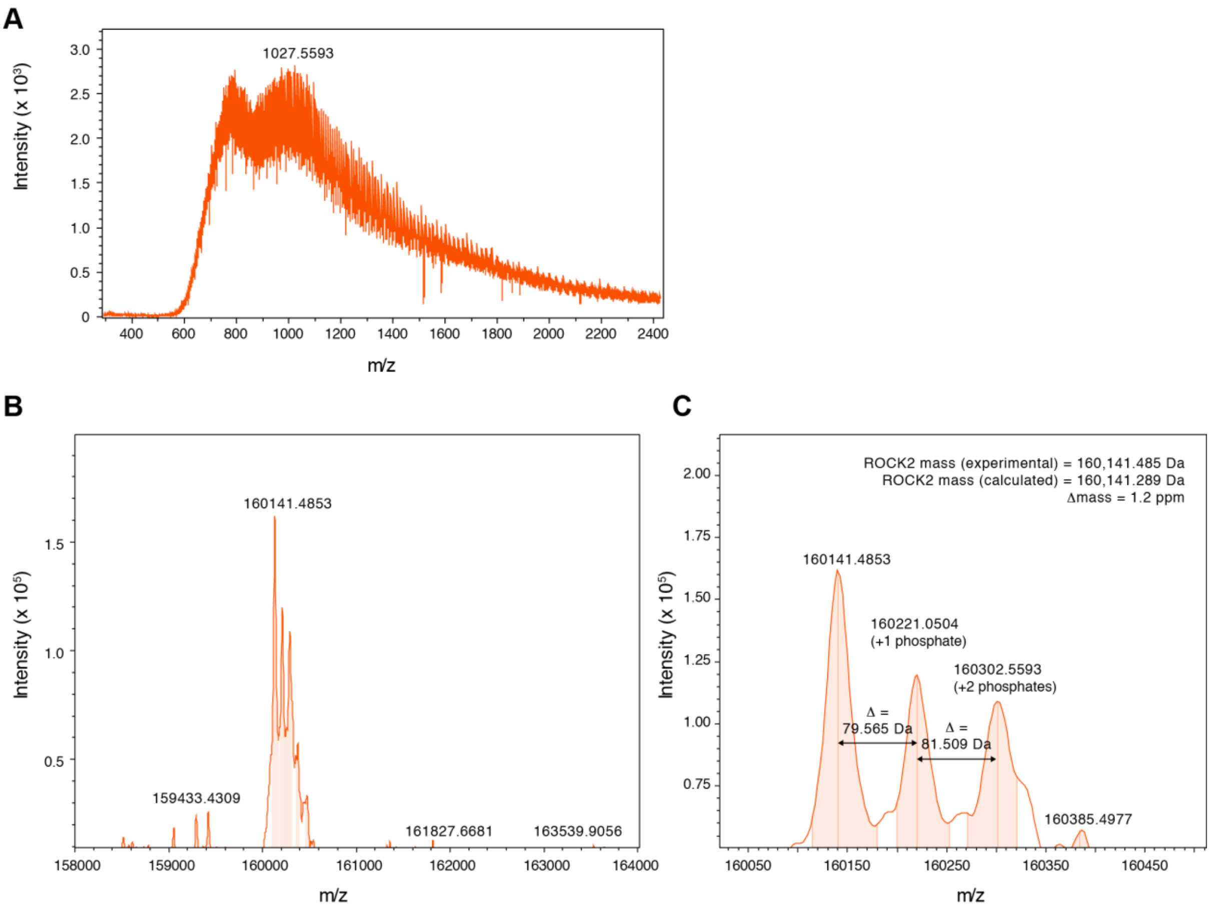

D

```
   1  MPGAPETAPG DGAGASRQRK LEALIRDPRS PINVESLLDG LNSLVLDLDF PALRKNKNID NFLNRYEKIV KKIRGLQMKA EDYDVVKVIG RGAFGEVQLV
 101  RHKASQKVYA MKLLSKFEMI KRSDSAFFWE ERDIMAFANS PWVVQLFYAF QDDRYLYMVM EYMPGGDLVN LMSNYDVPEK WAKFYTAEVV LALDAIHSMG
 201  LIHRDVKPDN MLLDKHGHLK LADFGTCMKM DETGMVHCDT AVGTPDYISP EVLKSQGGDG FYGRECDWWS VGVFLYEMLV GDTPFYADSL VGTYSKIMDH
 301  KNSLCFPEDA EISKHAKNLI CAFLTDREVR LGRNGVEEIR QHPFFKNDQW HWDNIRETAA PVVPELSSDI DSSNFDDIED DKGDVETFPI PKAFVGNQLP
 401  FIGFTYYREN LLLSDSPSCR ETDSIQSRKN EESQEIQKKL YTLEEHLSNE MQAKEELEQK CKSVNTRLEK TAKELEEEIT LRKSVESALR QLEREKALLQ
 501  HKNAEYQRKA DHEADKKRNL ENDVNSLKDQ LEDLKKRNQN SQISTEKVNQ LQRQLDETNA LLRTESDTAA RLRKTQAESS KQIQQLESNN RDLQDKNCLL
 601  ETAKLKLEKE FINLQSALES ERRDRTHGSE IINDLQGRIC GLEEDLKNGK ILLAKVELEK RQLQERFTDL EKEKSNMEID MTYQLKVIQQ SLEQEEAEHK
 701  ATKARLADKN KIYESIEEAK SEAMKEMEKK LLEERTLKQK VENLLLEAEK RCSLLDCDLK QSQQKINELL KQKDVLNEDV RNLTLKIEQE TQKRCLTQND
 801  LKMQTQQVNT LKMSEKQLKQ ENNHLMEMKM NLEKQNAELR KERQDADGQM KELQDQLEAE QYFSTLYKTQ VRELKEECEE KTKLGKELQQ KKQELQDERD
 901  SLAAQLEITL TKADSEQLAR SIAEEQYSDL EKEKIMKELE IKEMMARHKQ ELTEKDATIA SLEETNRTLT SDVANLANEK EELNNKLKDV QEQLSRLKDE
1001  EISAAAIKAQ FEKQLLTERT LKTQAVNKLA EIMNRKEPVK RGNDTDVRRK EKENRKLHME LKSEREKLTQ QMIKYQKELN EMQAQIAEES QIRIELQMTL
1101  DSKDSDIEQL RSQLQALHIG LDSSSIGSGP GDAEADDGFP ESRLEGWLSL PVRNNTKKFG WVKKYVIVSS KKILFYDSEQ DKEQSNPYMV LDIDKLFHVR
1201  PVTQTDVYRA DAKEIPRIFQ ILYANEGESK KEQEFPVEPV GEKSNYICHK GHEFIPTLYH FPTNCEACMK PLWHMFKPPP ALECRRCHIK CHKDHMDKKE
1301  EIIAPCKVYY DISTAKNLLL LANSTEEQQK WVSRLVKKIP KKPPAPDPFA RSSPRTSMKI QQNQSIRRPS RQLAPNKPS
```

E

Position	Localisation probability	PEP	Score	Score diff.	Mass error (ppm)	m/z	Intensity
240	0.95	1.00E-02	74.13	12.68	-0.43	949.0759	944900
244	1.00	9.03E-03	75.717	38.37	-0.46	949.0759	670190
418	0.51	8.40E-12	211.92	0.21	-0.32	796.3563	6651200
433	1.00	1.09E-03	136.81	136.81	1.19	720.8402	577260
1352	0.78	3.82E-03	124.46	5.38	0.46	534.9311	37754000
1353	0.80	1.14E-03	139.87	5.97	-0.23	801.8931	24704000

Supplementary Figure 1.

A. Intact mass spectrometry of recombinant ROCK2. Charge envelope of ROCK2.

B. Deconvoluted mass spectrum of ROCK2.

C. Zoom of deconvoluted mass spectrum showing the three predominant species corresponding to un-, mono-, and di-phosphorylated ROCK2. Unmodified ROCK2 was measured to a mass accuracy of 1.2 ppm. The presence of phosphate can be seen in the mass differences of 79.5 and 81.5 Da, which represents exceptional accuracy at this mass range.

D. Tryptic digest MS of recombinant ROCK2. Sequence coverage map of ROCK2. The two peptides covering the RhoA binding site are highlighted in green. Both unmodified peptides were detected, consistent with the intact mass measurements (C).

E. Phosphopeptides identified by MS/MS. pS1352 and pS1353 phosphopeptides (highlighted) were most abundant, and most likely correspond to the mono- and di-phosphorylated ROCK2 species observed by intact MS (C).

Supplementary Figure 2. ROCK2 adopts an extended, semi-rigid conformation in cells.

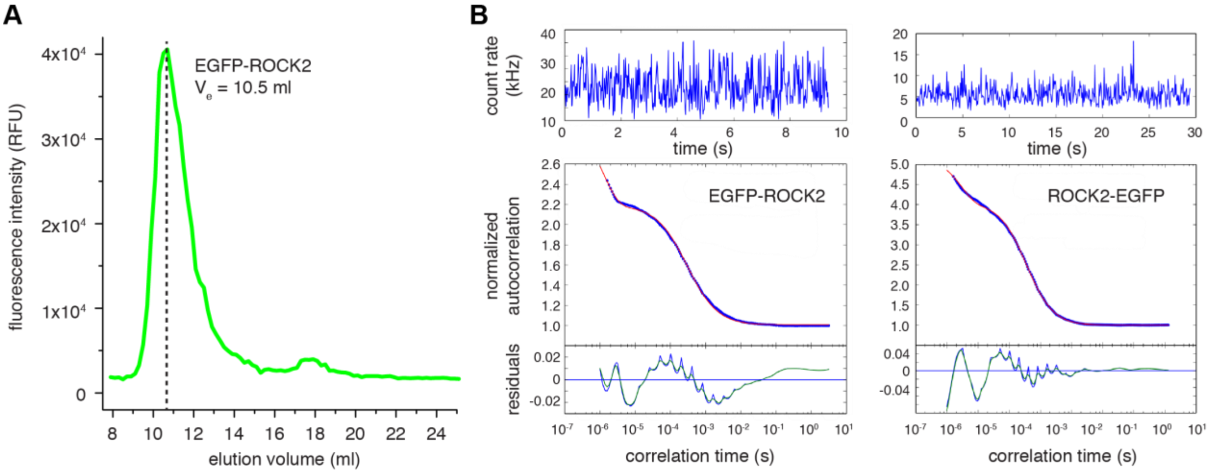

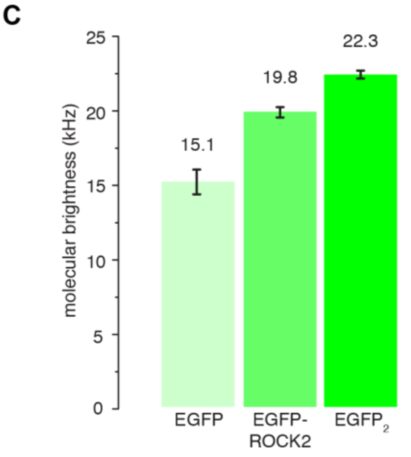

D

Protein	Diffusion coefficient (*D*) (x 10^{-11} m^2 s^{-1})	$D_{theor.}$ (globular) (x 10^{-11} m^2 s^{-1})	$D_{theor.}$ (rod) (x 10^{-11} m^2 s^{-1})	Apparent brightness (kHz)
EGFP	8.9 ± 0.2	10.3	-	15.1 ± 0.8
EGFP-ROCK2	2.7 ± 0.1	*4.3	1.8 x 10^{-11}	19.8 ± 0.3
$EGFP_2$	6.4 ± 0.1	8.2	-	22.3 ± 0.3
EGFP	9.0 ± 0.5	10.3	-	15.8 ± 0.4
ROCK2-EGFP	2.5 ± 0.1	*4.3	1.8 x 10^{-11}	17.3 ± 0.8

*calculated for a dimer of ROCK2 (320 kDa)

Supplementary Figure 2.

A. Fluorescence size exclusion chromatography of ROCK2-EGFP expressing COS7 cell lysates (Superose 6).

B. Fluorescence correlation spectroscopy of the peak fraction containing EGFP-tagged ROCK2. Both N- and C-terminal fusions were best fitted with a single diffusion component for which the residuals are shown.

C. Molecular brightness of ROCK2. ROCK2 expressed in COS7 fibroblasts exhibits a molecular brightness intermediate between EGFP and a tandem EGFP ($EGFP_2$) construct.

D. Table comparing the measured diffusion coefficients with calculated diffusion coefficients for both a globular protein and a stiff rod of the dimensions observed by electron microscopy of the recombinant protein.

Supplementary Figure 3 (A-E). ROCK2 function depends on the length of its coiled-coil.

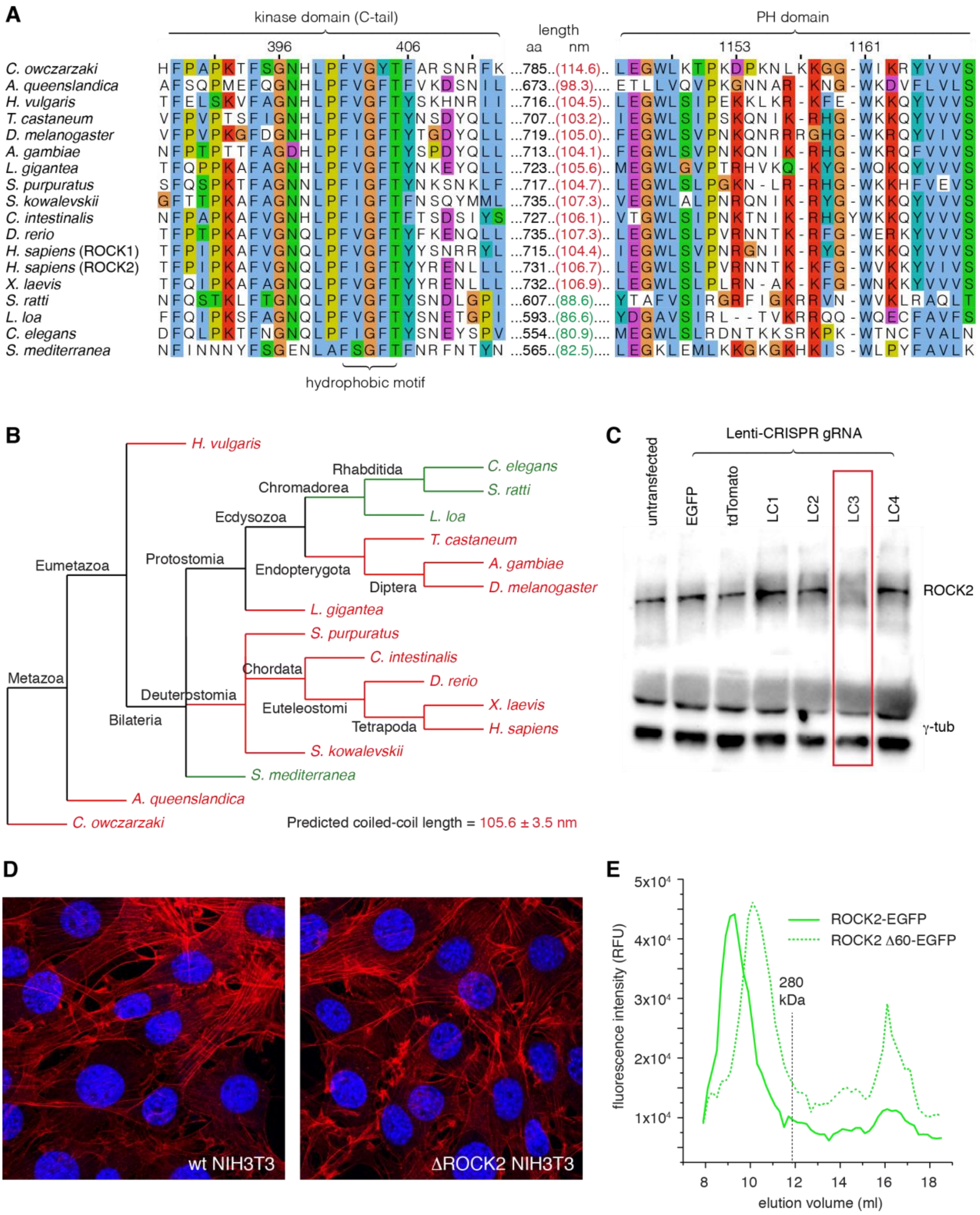

Supplementary Figure 3 (F).

F

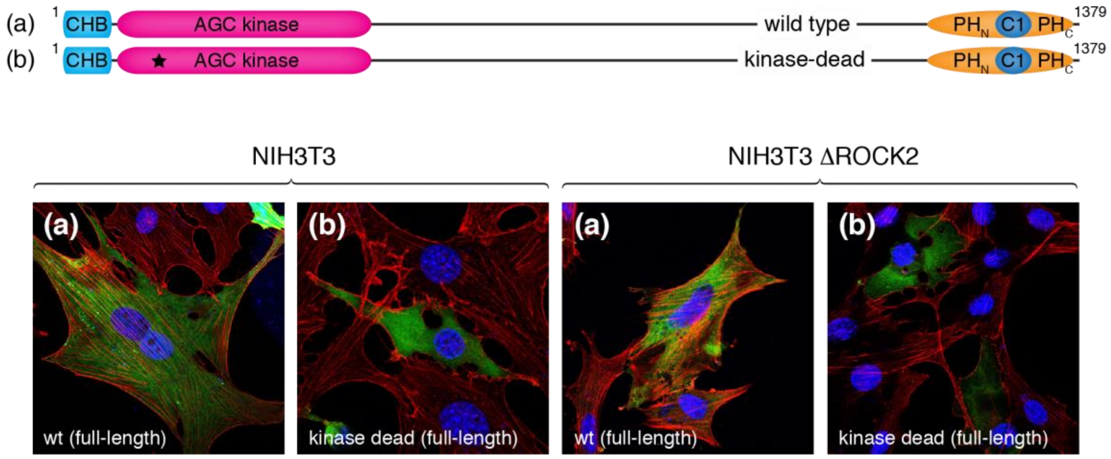

Supplementary Figure 3.

A. Alignment of ancestral ROCK proteins. The boundaries of the inter-domain coiled coil spacer were determined from a multiple sequence alignment of ROCK orthologues from 17 species spanning evolutionary time from *Capsaspora owczarzaki* to *Homo sapiens*. Representative parts of the full sequence alignment covering the end of the AGC kinase domain and the beginning of the PH domain are shown (left and right, respectively). The last amino acid of the C-terminal tail of human ROCK2 was assigned to be residue 413 and the first amino acid of the PH domain was assigned to be residue 1145. The number of intervening amino acids was calculated for each protein based on these boundaries and is shown between the two alignments (black). The corresponding length of a canonical coiled coil (1.46 Å rise/amino acid) of the calculated number of amino acids is shown alongside in parentheses (red/green). The alignment numbering corresponds to human ROCK2 and the conserved hydrophobic motif that characterizes the C-terminal tail of these kinases is annotated.

B. Phylogenetic tree of the organisms represented by sequences in A. Species marked in red correspond to those with coiled coils clustering around 105 nm in length, while those in green cluster around 85 nm. The earliest record of ROCK can be found in the unicellular eukaryote *C. owkzarzaki*.

C. CRISPR/Cas9 knockout of ROCK2 in NIH3T3 cells. Control guide RNAs for EGFP and tdTomato have no effect on ROCK2 in lentivirus-transduced cells. Of four guide RNAs tested for ROCK2 knockout, LC3 resulted in a complete knockout of ROCK2.

D. Immunostaining of wild type 3T3 and ΔROCK2 3T3 fibroblasts. The actin cytoskeleton of the knockout cells, including the density and distribution of stress fibers, appears morphologically indistinct from the wild type cells.

E. Fluorescence size exclusion chromatography of ROCK2 Δ60 nm (280 kDa) in NIH3T3 ΔROCK2 fibroblast lysates. ROCK2 Δ60 nm elutes as a single species with an elution volume consistent with an extended particle 60 nm in length (expected elution volume for globular protein of 280 kDa shown for comparison).

F. Expression phenotypes of $ROCK2^{wt}$ and $ROCK2^{KD}$ in NIH3T3 and ΔROCK2 NIH3T3 fibroblasts. Expression of $ROCK2^{wt}$ supports normal stress fibers, while expression of kinase-dead $ROCK2^{KD}$ results in complete loss of stress fibers in both cases.

Supplementary Figure 4 (A-D). ROCK2 does not bind to and is not activated by RhoA.

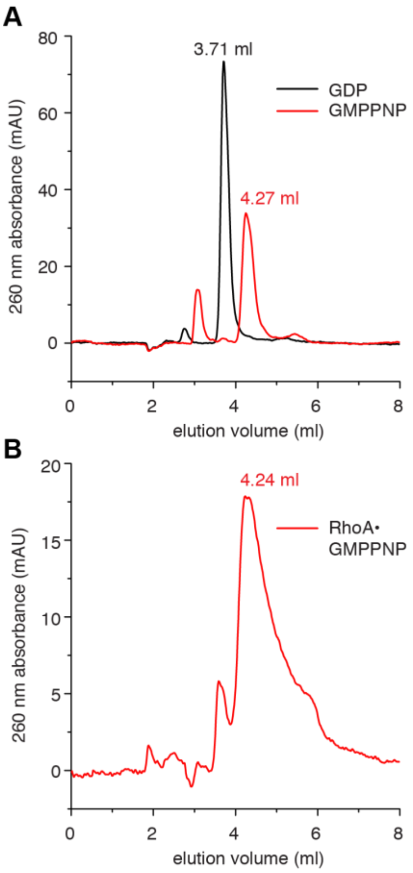

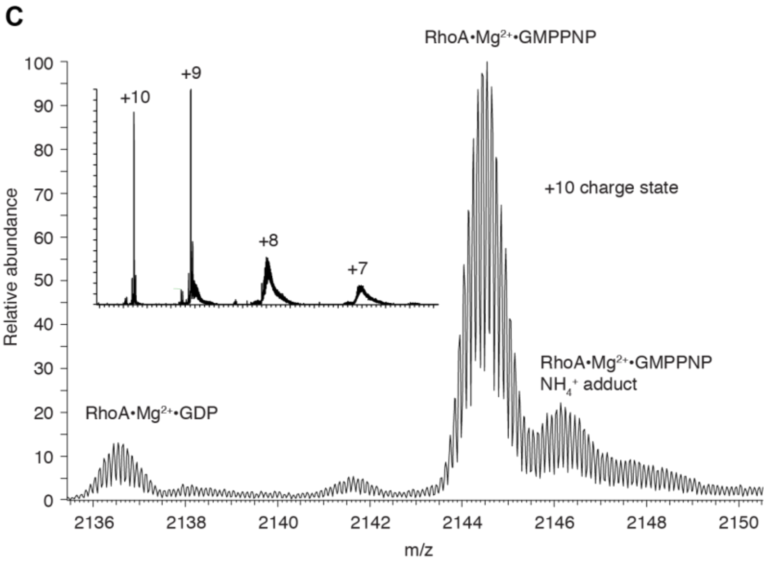

Protein:ligand complex	Theoretical average mass (Da)	Observed average mass (Da)	Standard deviation (Da)	Delta (Da)
RhoA•Mg^{2+}•GDP	21356.0187	21356.0745	0.0443	0.0558
RhoA•Mg^{2+}•GDP•NH_4^+	21373.0452	21373.7913	n.d.	0.7461
RhoA•Mg^{2+}•GMPPNP	21435.0137	21435.1440	0.0269	0.1304
RhoA•Mg^{2+}•GMPPNP•NH_4^+	21453.0242	21452.2389	0.6417	-0.7853

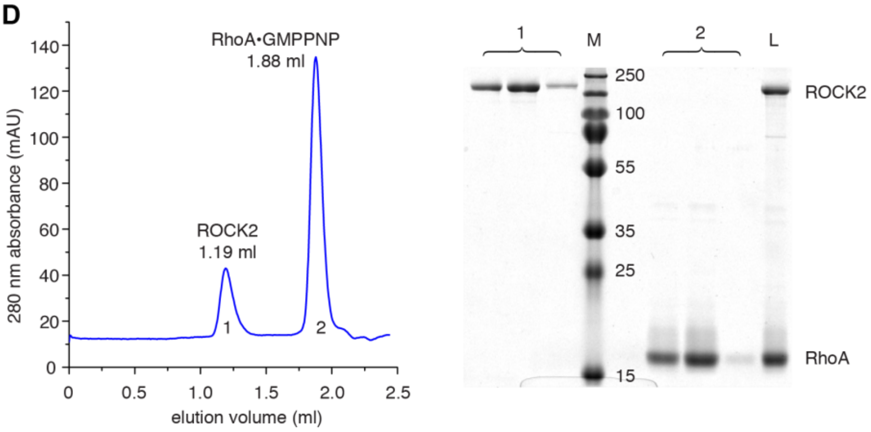

Supplementary Figure 4 (E-I).

E

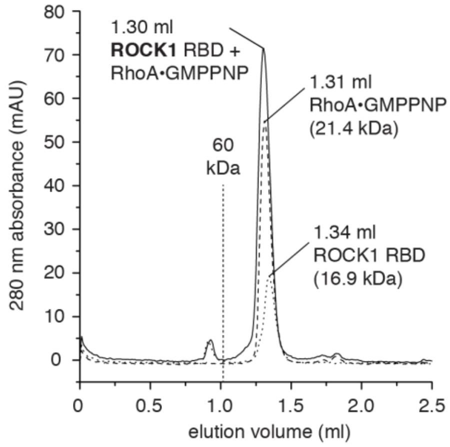

F

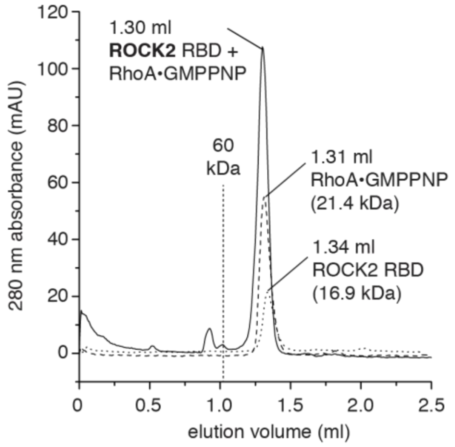

G

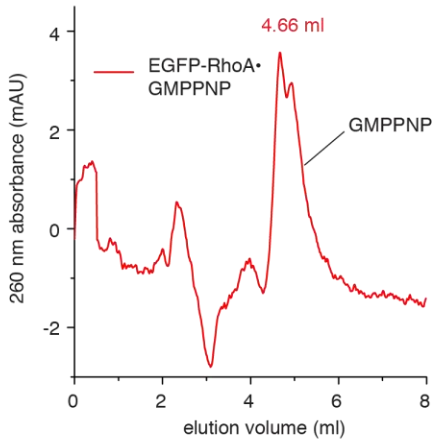

H

	LEU	TRP	Fusion partner A	Fusion partner B	Signal
1	pDP177	pDP134	GAD	LexA	no
2	pDP177-RhoA	pDP135-RBD	GAD-RhoA	LexA-RBD	no
3	pDP177-RBD	pDP135-RhoA	GAD-RBD	LexA-RhoA	weak
4	pDP177-RhoA	pDP135	GAD-RhoA	LexA	no
5	pDP177-RBD	pDP135	GAD-RBD	LexA	no
6	pDP177	pDP135-RhoA	GAD	LexA-RhoA	no
7	pDP177	pDP135-RBD	GAD	LexA-RBD	no
8	pDP176	pDP167	Atg13-MIM-GAD	LexA-Atg1	yes
9	pDP177-ROCK1	pDP135-RhoA	GAD-ROCK1	LexA-RhoA	no
10	pDP177-ROCK2	pDP135-RhoA	GAD-ROCK2	LexA-RhoA	no
11	pDP177-RhoA	pDP135-ROCK1	GAD-RhoA	LexA-ROCK1	no
12	pDP177-RhoA	pDP135-ROCK2	GAD-RhoA	LexA-ROCK2	no
13	pDP177-ROCK1	pDP135	GAD-ROCK1	LexA	no
14	pDP177-ROCK2	pDP135	GAD-ROCK2	LexA	no
15	pDP177-RhoA	pDP135	GAD-RhoA	LexA	no
16	pDP177	pDP135-ROCK1	GAD	LexA-ROCK1	no
17	pDP177	pDP135-ROCK2	GAD	LexA-ROCK2	no
18	pDP177	pDP135-RhoA	GAD	LexA-RhoA	no
19	pCK841	pCK846	GAD-Atg17	LexA-Atg13$^{424-436}$	yes
20	pDP177	pDP135	GAD	LexA	no

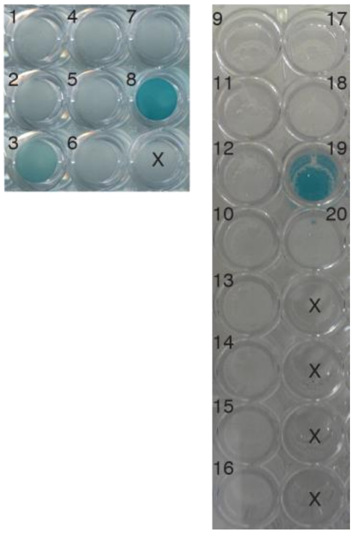

I

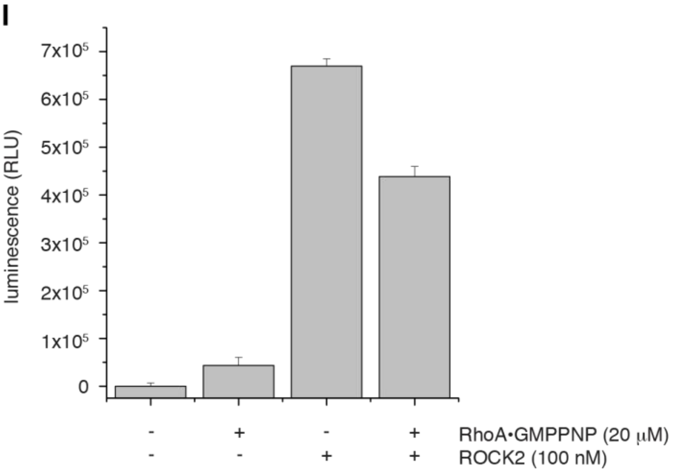

Supplementary Figure 4.

A. Ion-pairing reverse-phase HPLC. GDP and GMPPNP standards separated in a mobile phase of 100 mM KH_2PO_4/K_2HPO_4 pH 6.5, 10 mM tetra-n-butylammonium bromide, 4.5 % CH_3CN on a C18 reverse phase column.

B. Confirmation of RhoA loading with GMPPNP. Ion-pairing reverse HPLC of nucleotide-exchanged RhoA.

C. Native mass spectrometry demonstrates the loading of RhoA with GMP-PNP. Mass spectrum of the +10 charge state showing the isotopic envelope of RhoA loaded with GDP and GMPPNP, respectively. The inset depicts an overview of all recorded charge states. The table below indicates the theoretical and observed masses.

D. Size exclusion chromatography of ROCK2 + RhoA. A 1:10 mixture of ROCK2:RhoA•GMPPNP was incubated for 30 min at 4°C and separated by size exclusion chromatography on a Superose 6 Increase 3.2/300 gel filtration column equilibrated in 20 mM Tris, pH 7.5, 150 mM KCl, 1 mM TCEP, 4 mM $MgCl_2$. ROCK2 and RhoA eluted as separate peaks at elution volumes identical to the proteins when run alone. Since the anomalous elution volume of ROCK2 might not be expected to change significantly upon the addition of RhoA, the peak fractions were analyzed by SDS-PAGE (right). As can be seen, ROCK2 is found exclusively in the first peak, while RhoA is found exclusively in the second peak, indicating that a stable complex does not form between the two proteins under these conditions. (M = marker, L = load).

E. Size exclusion chromatography of ROCK1 Rho-binding domain (RBD) fragments with RhoA. A 2:1 mixture of ROCK1 RBD (945-1015):RhoA•GMPPNP was incubated for 30 min at 4°C and separated by size exclusion chromatography on

a Superdex 75 3.2/300 gel filtration column equilibrated in 20 mM Tris, pH 7.5, 150 mM KCl, 1 mM TCEP, 4 mM $MgCl_2$ (physiological salt concentration) or 20 mM Tris, pH 7.5, 1 mM TCEP, 4 mM $MgCl_2$ (zero salt). ROCK1 RBD and RhoA co-eluted at an elution volume of ~1.3 ml consistent with the elution volumes of the proteins alone and a molecular weight of ~20 kDa. Curves shown are for physiological salt concentration; elution profiles in zero salt were identical.

F. Size exclusion chromatography of ROCK2 Rho-binding domain (RBD) fragments with RhoA. A 2:1 mixture of ROCK2 RBD (968-1038):RhoA•GMPPNP was incubated for 30 min at 4°C and separated by size exclusion chromatography on a Superdex 75 3.2/300 gel filtration column equilibrated in 20 mM Tris, pH 7.5, 150 mM KCl, 1 mM TCEP, 4 mM $MgCl_2$ (physiological salt concentration) or 20 mM Tris, pH 7.5, 1 mM TCEP, 4 mM $MgCl_2$ (zero salt). ROCK1 RBD and RhoA co-eluted at an elution volume of ~1.3 ml consistent with the elution volumes of the proteins alone and a molecular weight of ~20 kDa. Curves shown are for physiological salt concentration; elution profiles in zero salt were identical.

G. Ion-pairing reverse-phase HPLC confirms loading of EGFP-RhoA with GMPPNP.

H. Y2H analysis of the interaction of RhoA with ROCK1 and ROCK2. The results of the liquid assay interaction screening are shown. The table details the individual fusion partners in each experiment. For completeness, we tested each combination of putative interaction partners twice, where in the second experiment the GAD and LexA domains have been switched. We observed strong signal for both positive controls (blue), but no signal for the interaction of ROCK1 or ROCK2 with RhoA. We observed a weak signal for the interaction of GAD-RBD (ROCK1) with LexA-RhoA (3), but this signal was not observed when the GAD

and LexA domains were switched (2). Protein expression was confirmed in each case by immunoblot against the GAD or LexA domain.

I. ROCK2 kinase assay in the presence of RhoA. ROCK2 was incubated in the absence and presence of a 200-fold excess of activated RhoA (RhoA•GMPPNP) under conditions of non-limiting substrate and ATP. Kinase activity was normalized to a no-kinase control. Excess RhoA results in a 35% reduction in ROCK2 activity.

Supplementary Figure 5. ROCK is a constitutively active kinase independent of activation loop phosphorylation.

A

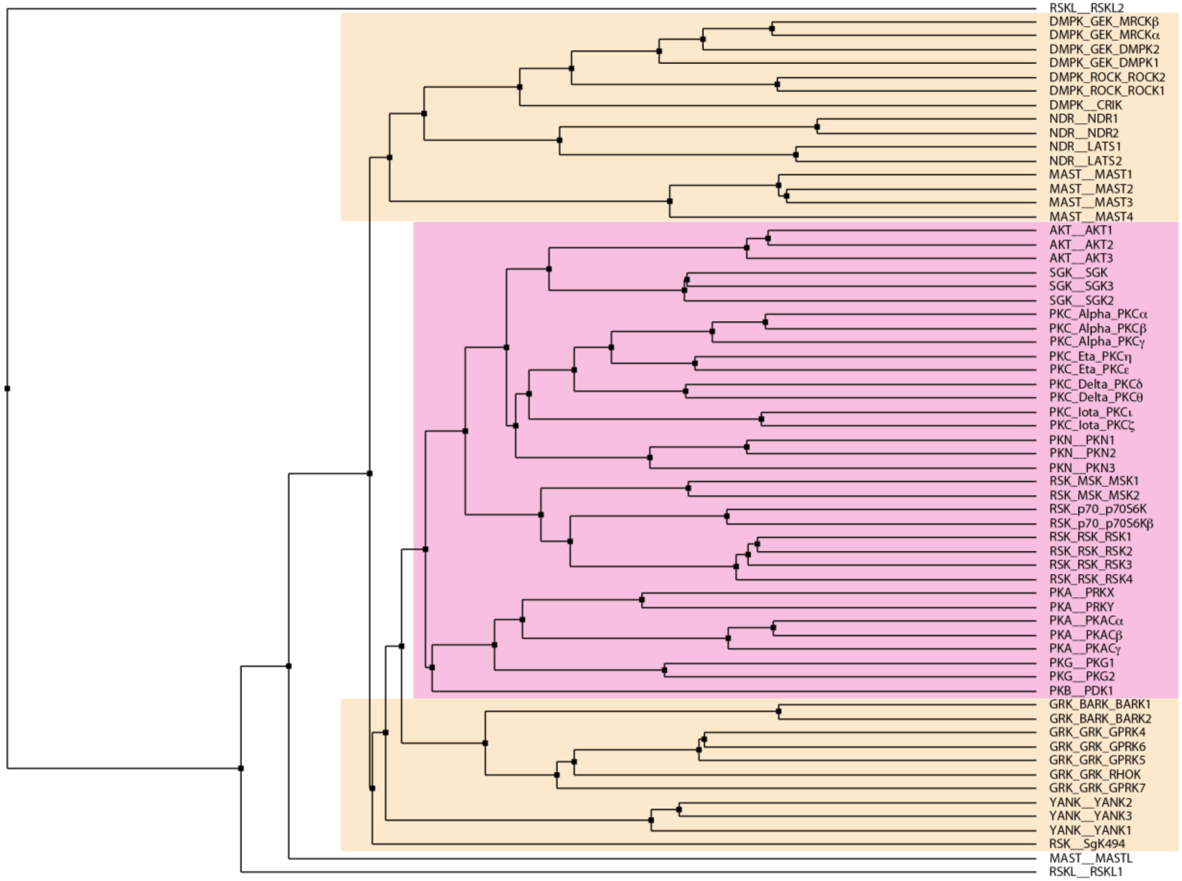

ACTIVATION LOOP

Kinase	Activation loop	Mark	Group
RSKL__RSKL2	QCCGEAVDNLYSAP		
DMPK_GEK_MRCKβ	QSSVAVGTPDYISP	•	UNPHOSPHORYLATED
DMPK_GEK_MRCKα	QSSVAVGTPDYISP	•	UNPHOSPHORYLATED
DMPK_GEK_DMPK2	DSSVAVGTPDYISP		UNPHOSPHORYLATED
DMPK_GEK_DMPK1	RSLVAVGTPDYLSP		UNPHOSPHORYLATED
DMPK_ROCK_ROCK2	HCDTAVGTPDYISP	•	UNPHOSPHORYLATED
DMPK_ROCK_ROCK1	RCDTAVGTPDYISP	•	UNPHOSPHORYLATED
DMPK__CRIK	NAKLPIGTPDYMAP		UNPHOSPHORYLATED
NDR__NDR1	LAFSTVGTPDYIAP		UNPHOSPHORYLATED
NDR__NDR2	LAYSTVGTPDYIAP		UNPHOSPHORYLATED
NDR__LATS1	LAHSLVGTPNYIAP		UNPHOSPHORYLATED
NDR__LATS2	LAHSLVGTPNYIAP		UNPHOSPHORYLATED
MAST__MAST1	LDKQVCGTPEYIAP		UNPHOSPHORYLATED
MAST__MAST2	LDKQVCGTPEYIAP		UNPHOSPHORYLATED
MAST__MAST3	IDKQVCGTPEYIAP		UNPHOSPHORYLATED
MAST__MAST4	LDKQVCGTPEYIAP		UNPHOSPHORYLATED
AKT__AKT1	TMKTFCGTPEYLAP	*	PHOSPHORYLATED
AKT__AKT2	TMKTFCGTPEYLAP	*	PHOSPHORYLATED
AKT__AKT3	TMKTFCGTPEYLAP		PHOSPHORYLATED
SGK__SGK	TTSTFCGTPEYLAP		PHOSPHORYLATED
SGK__SGK3	TTTTFCGTPEYLAP		PHOSPHORYLATED
SGK__SGK2	TTSTFCGTPEYLAP		PHOSPHORYLATED
PKC_Alpha_PKCα	TTRTFCGTPDYIAP		PHOSPHORYLATED
PKC_Alpha_PKCβ	TTKTFCGTPDYIAP	*	PHOSPHORYLATED
PKC_Alpha_PKCγ	TTRTFCGTPDYIAP		PHOSPHORYLATED
PKC_Eta_PKCη	TTATFCGTPDYIAP		PHOSPHORYLATED
PKC_Eta_PKCε	TTTTFCGTPDYIAP		PHOSPHORYLATED
PKC_Delta_PKCδ	RASTFCGTPDYIAP		PHOSPHORYLATED
PKC_Delta_PKCθ	KTNTFCGTPDYIAP		PHOSPHORYLATED
PKC_Iota_PKCι	TTSTFCGTPNYIAP	*	PHOSPHORYLATED
PKC_Iota_PKCζ	TTSTFCGTPNYIAP		PHOSPHORYLATED
PKN__PKN1	RTSTFCGTPEFLAP		PHOSPHORYLATED
PKN__PKN2	RTSTFCGTPEFLAP		PHOSPHORYLATED
PKN__PKN3	RTSTFCGTPEFLAP		PHOSPHORYLATED
RSK_MSK_MSK1	RAYSFCGTIEYMAP		PHOSPHORYLATED
RSK_MSK_MSK2	RTFSFCGTIEYMAP		PHOSPHORYLATED
RSK_p70_p70S6K	VTHTFCGTIEYMAP		PHOSPHORYLATED
RSK_p70_p70S6Kβ	VTHTFCGTIEYMAP		PHOSPHORYLATED
RSK_RSK_RSK1	RAYSFCGTIEYMAP	**	PHOSPHORYLATED
RSK_RSK_RSK2	KAYSFCGTVEYMAP		PHOSPHORYLATED
RSK_RSK_RSK3	KAYSFCGTVEYMAP		PHOSPHORYLATED
RSK_RSK_RSK4	KAYSFCGTVEYMAP		PHOSPHORYLATED
PKA__PRKX	RTWTLCGTPEYLAP	*	PHOSPHORYLATED
PKA__PRKY	RTWTLCGTPEYLAP		PHOSPHORYLATED
PKA__PKACα	RTWTLCGTPEYLAP	*	PHOSPHORYLATED
PKA__PKACβ	RTWTLCGTPEYLAP		PHOSPHORYLATED
PKA__PKACγ	RTWTLCGTPEYLAP		PHOSPHORYLATED
PKG__PKG1	KTWTFCGTPEYVAP	*	PHOSPHORYLATED
PKG__PKG2	KTWTFCGTPEYVAP		PHOSPHORYLATED
PKB__PDK1	RANSFVGTAQYVSP	*	PHOSPHORYLATED
GRK_BARK_BARK1	KPHASVGTHGYMAP	•	UNPHOSPHORYLATED
GRK_BARK_BARK2	KPHASVGTHGYMAP	•	UNPHOSPHORYLATED
GRK_GRK_GPRK4	RVRGRVGTVGYMAP		UNPHOSPHORYLATED
GRK_GRK_GPRK5	LIRGRVGTVGYMAP	•	UNPHOSPHORYLATED
GRK_GRK_GPRK6	TIKGRVGTVGYMAP		UNPHOSPHORYLATED
GRK_GRK_RHOK	KTKGYAGTPGFMAP		UNPHOSPHORYLATED
GRK_GRK_GPRK7	PITQRAGTNGYMAP		UNPHOSPHORYLATED
YANK__YANK2	RASSMAGTKPYMAP		UNPHOSPHORYLATED
YANK__YANK1	QITTMAGTKPYMAP	•	UNPHOSPHORYLATED
YANK__YANK3	RATALAGTKPYMAP		UNPHOSPHORYLATED
RSK__SgK494	QAYTICGTLQYMAP		UNPHOSPHORYLATED
MAST__MASTL	NMMDILTTPSMAKP		
RSKL__RSKL1	SCDSDAIERMYCAP		

- • ordered, active conformation, unphosphorylated activation loop (ROCK1, 2V55; ROCK2, 2F2U; MRCKα, 4AW2; MRCKβ, 3TKU; GRK1, 3C4W; GRK2, 2BCJ; GRK6, 2ACX; YANK, 4FR4)
- * ordered, active conformation, phosphorylated activation loop
- ** disordered, inactive conformation, unphosphorylated activation loop

Supplementary Figure 5B-C.

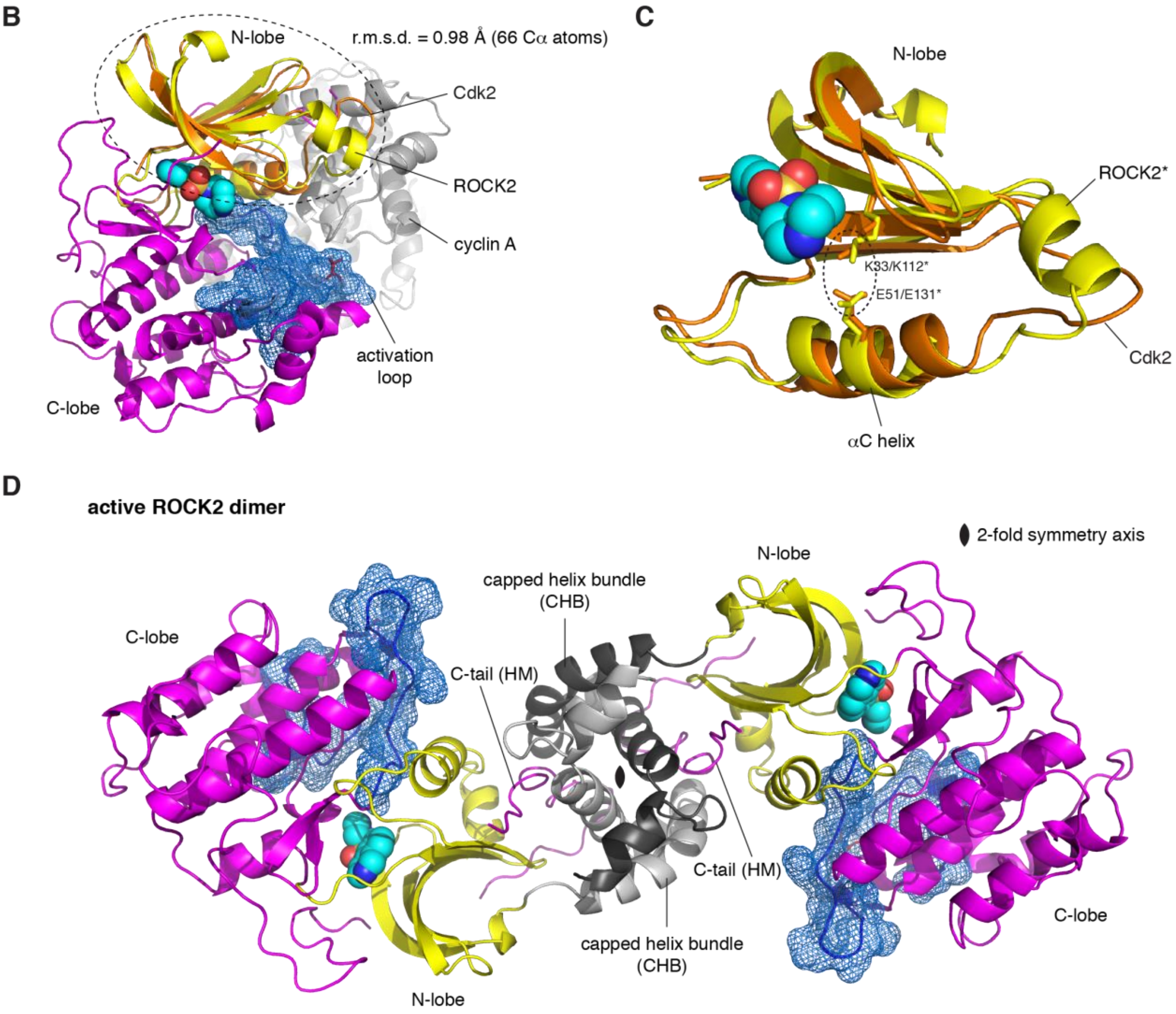

Supplementary Figure 5.

A. Alignment of the activation loop of all AGC kinases. AGC kinases that conserve a phosphorylatable threonine or serine in their activation loop at the canonical position that has been shown to be important for activity belong to a single branch of the AGC kinome tree (magenta). Structural data exists for 9 of 34 kinases in this branch in which the activation loop is disordered in its unphosphorylated state (**), or ordered in its phosphorylated state (*). The remaining AGC kinases do not conserve a phosphorylatable residue at the

canonical position (yellow). Structural data exists for 8 of 29 kinases in which the activation loop adopts an ordered, active conformation in the absence of phosphorylation (•).

B. Superposition of the N-lobes of ROCK2 (2F2U, 91-172) and the active conformation of Cdk2 in complex with cyclin A (1FIN, chain A, 3-83). The N-lobes show an r.m.s.d. of just 0.98 Å over 66 Cα atoms.

C. Superposition of the N-lobes of ROCK2 and Cdk2, illustrating the conserved salt bridge between a glutamate in the αC helix and a lysine in strand β3 that characterizes the active conformation of protein kinases.

D. The active dimer of ROCK2. The CHB domain (grey) combines with the C-terminal tail of the kinase domain, in particular the hydrophobic motif (HM), to stabilize the active conformation of the N-lobe, analogous to the role performed by cyclin A with Cdk2. The activation loops of each monomer are fully ordered in the absence of phosphorylation.
